# Application of machine learning in the diagnosis of gastric cancer based on noninvasive characteristics

**DOI:** 10.1371/journal.pone.0244869

**Published:** 2020-12-31

**Authors:** Shuang-Li Zhu, Jie Dong, Chenjing Zhang, Yao-Bo Huang, Wensheng Pan

**Affiliations:** 1 Department of Geriatric VIP NO.1, Zhejiang Provincial People’s Hospital, People’s Hospital of Hangzhou Medical College, Hangzhou, Zhejiang Province, China; 2 Department of Gastroenterology, Zhejiang Provincial People’s Hospital, People’s Hospital of Hangzhou Medical College, Hangzhou, Zhejiang Province, China; 3 Department of Financial Security, Alibaba Group, Hangzhou, Zhejiang Province, China; Indiana University School of Medicine, UNITED STATES

## Abstract

**Background:**

The diagnosis of gastric cancer mainly relies on endoscopy, which is invasive and costly. The aim of this study is to develop a predictive model for the diagnosis of gastric cancer based on noninvasive characteristics.

**Aims:**

To construct a predictive model for the diagnosis of gastric cancer with high accuracy based on noninvasive characteristics.

**Methods:**

A retrospective study of 709 patients at Zhejiang Provincial People's Hospital was conducted. Variables of age, gender, blood cell count, liver function, kidney function, blood lipids, tumor markers and pathological results were analyzed. We used gradient boosting decision tree (GBDT), a type of machine learning method, to construct a predictive model for the diagnosis of gastric cancer and evaluate the accuracy of the model.

**Results:**

Of the 709 patients, 398 were diagnosed with gastric cancer; 311 were health people or diagnosed with benign gastric disease. Multivariate analysis showed that gender, age, neutrophil lymphocyte ratio, hemoglobin, albumin, carcinoembryonic antigen (CEA), carbohydrate antigen 125 (CA125) and carbohydrate antigen 199 (CA199) were independent characteristics associated with gastric cancer. We constructed a predictive model using GBDT, and the area under the receiver operating characteristic curve (AUC) of the model was 91%. For the test dataset, sensitivity was 87.0% and specificity 84.1% at the optimal threshold value of 0.56. The overall accuracy was 83.0%. Positive and negative predictive values were 83.0% and 87.8%, respectively.

**Conclusion:**

We construct a predictive model to diagnose gastric cancer with high sensitivity and specificity. The model is noninvasive and may reduce the medical cost.

## Introduction

Gastric cancer is a common malignancy with high incidence and mortality rates. Ranking third among cancers worldwide, the mortality of gastric cancer is 8.2% [1]. It has also been identified as one of the leading causes of cancer death in China [2]. Indeed, gastric cancer remains a serious health issue in China: there are approximately 400 000 new cases of gastric cancer in China every year, with a crude incidence rate of 42 per 100 000 in males and 20 per 100 000 in females. As the incidence and mortality rates of stomach cancer in urban areas are higher than those in rural areas [3, 4], prevention and control strategies should be implemented while considering regional differences.

At present, the diagnosis of gastric cancer mainly relies on endoscopy and surgery. However, some patients, especially in rural areas, refuse to undergo endoscopy or surgery due to the invasive nature and costliness of the procedure [5]. Unfortunately, there are no noninvasive characteristics defined for detecting gastric cancer with high sensitivity and specificity. Early diagnosis and early treatment are crucial for improving the survival rate and reducing the mortality of gastric cancer. Therefore, it is of great significance to explore noninvasive characteristics or models by which to diagnose gastric cancer.

Machine learning has been applied to detect many types of cancer, such as colorectal cancer [6] and breast cancer [7], with high accuracy. In the field of gastric cancer, machine learning is mainly applied for analyzing endoscopic images, which are obtained through invasive procedures [8, 9]. In contrast, detection of routine blood, biochemical, and tumor markers is noninvasive and inexpensive. Therefore, we constructed a predictive model using machine learning to diagnose gastric cancer based on these noninvasive characteristics.

## Materials and methods

The study was reviewed and approved by the institutional review board (IRB) of the Zhejiang Provincial People’s Hospital (220QT111). The need to obtain written informed consent for participants’ clinical records to be used was also waived by the IRB for this retrospective study. As was approved by the IRB, we used patient identification numbers to collect and analyze clinical records. Names and other personal information were anonymized and de-identified prior to analysis to protect patient privacy.

### Study subjects

We reviewed the medical records of 960 patients who were diagnosed with gastric cancer, benign gastric disease or health people from December 2018 to August 2019 at Zhejiang Provincial People's Hospital. We used medical record numbers to identify individual subjects. The inclusion criteria were as follows: i) age >18 years, ii) histologically confirmed gastric cancer, benign gastric disease or health, iii) complete relevant data, iv) no other cancer. The exclusion criteria included the following: i) incomplete relevant data, ii) double cancers, and iii) recurrent gastric cancer. Notably, 32 patients with another cancer and 166 with recurrent gastric cancer were excluded; 53 patients who had insufficient data were also excluded. Finally, 709 patients were enrolled in our study to develop a predictive model. The hypothesis of this study is that the predictive model can effectively distinguish gastric cancer patients from non-gastric cancer controls. We learned that the sensitivity of the predictive model was 0.8 and the specificity was 0.75 based on preliminary data from our early observation of 80 subjects (40 of each group). We used PASS software (PASS, version 11.0) to estimate the sample size. It was found that at least 23 patients in each group were required, with a two-tailed test of α = 0.05, 1 –β (the power) = 0.90 and the ratio between groups 1:1 (prevalence rate 0.5). Considering a 10% loss of drop-out rate, at least 26 patients in each group were required. Thus the sample size of this study met with the requirement. We conducted this study from August 2019 to March 2020.

The medical records of each patient were reviewed for noninvasive characteristics and pathological results. The noninvasive characteristics included age, gender, neutrophil count, lymphocyte count, neutrophil lymphocyte ratio(NLR), hemoglobin(Hb), red cell distribution width(RDW), platelet(Plt), albumin(Alb), alanine transaminase(ALT), total bilirubin(TB), creatinine(Cr),triglyceride(TG), high-density lipoprotein cholesterol(HDL-C), low-density lipoprotein cholesterol(LDL-C), lipoprotein(Lpa), carcinoembryonic antigen(CEA), carbohydrate antigen 125(CA125), carbohydrate antigen 199(CA199) and carbohydrate antigen 724(CA724). Pathological results were divided into gastric cancer, benign gastric disease or health. Gastric cancer included adenocarcinoma, in situ carcinoma, malignant gastrointestinal stromal tumor, signet ring cell carcinoma, non-Hodgkin lymphoma, papillary carcinoma and tubular adenocarcinoma. Benign gastric disease included benign gastrointestinal stromal tumor, lipoma, leiomyoma, neurilemmoma, fibroxanthoma and gland polyps. Healthy people were defined as no obvious abnormalities in pathological examination.

### Data preparation

We identified outliers, treated them as missing values and filled them by k-means clustering. A univariate analysis was performed to evaluate the relationship between the noninvasive characteristics and diagnosis of gastric cancer. Measurement data were treated with the t-test if they followed the normal distribution or treated with the Mann-Whitney U test if they did not follow the normal distribution. Enumeration data were treated with the chi-square test. Significant characteristics were screened in the univariate analysis. Then, a multivariate analysis was performed using the significant characteristics to screen independent characteristics for diagnosing gastric cancer. Multivariable analysis was performed by logistic regression. A P-value of less than 0.05 was considered to be significant. Data were analyzed with SPSS software (SPSS, version 26.0, United States).

**Fig 1:**
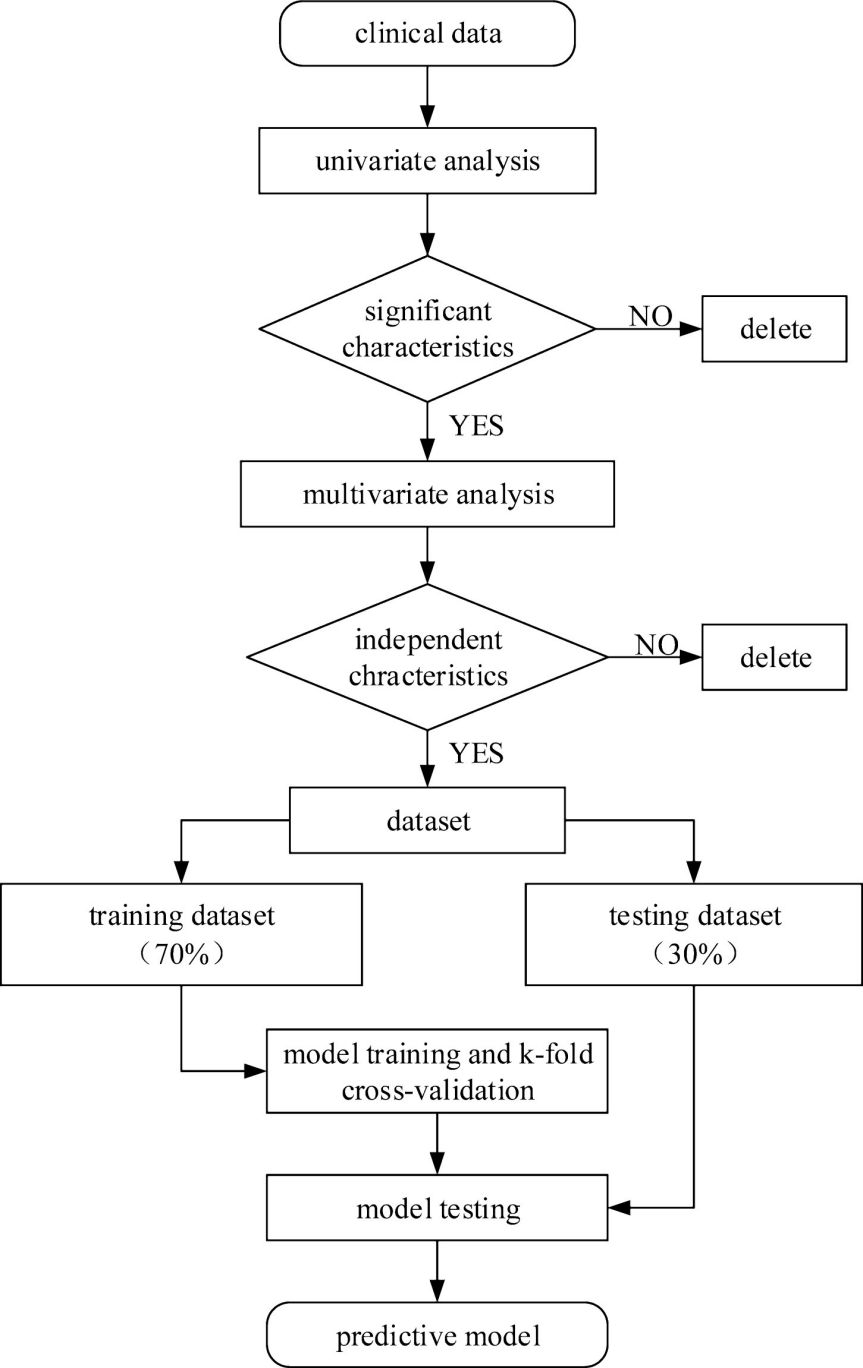
The procedure of data preparation and model construction. A univariate analysis was performed to evaluate the relationship between the characteristics and diagnosis of gastric cancer. Significant characteristics were screened in the univariate analysis. Then, a multivariate analysis was performed to screen independent characteristics for diagnosing gastric cancer. Independent characteristics related to gastric cancer were selected to construct a dataset. The dataset was randomly divided into a training dataset to construct the model and a test dataset to test the model.

Independent characteristics related to gastric cancer were selected to construct a dataset. The dataset was randomly divided into a training dataset (n = 496) and a test dataset (n = 213) with a proportion of 7:3. The procedure of our study is shown in Fig 1.

### Training algorithm

**Fig 2:**
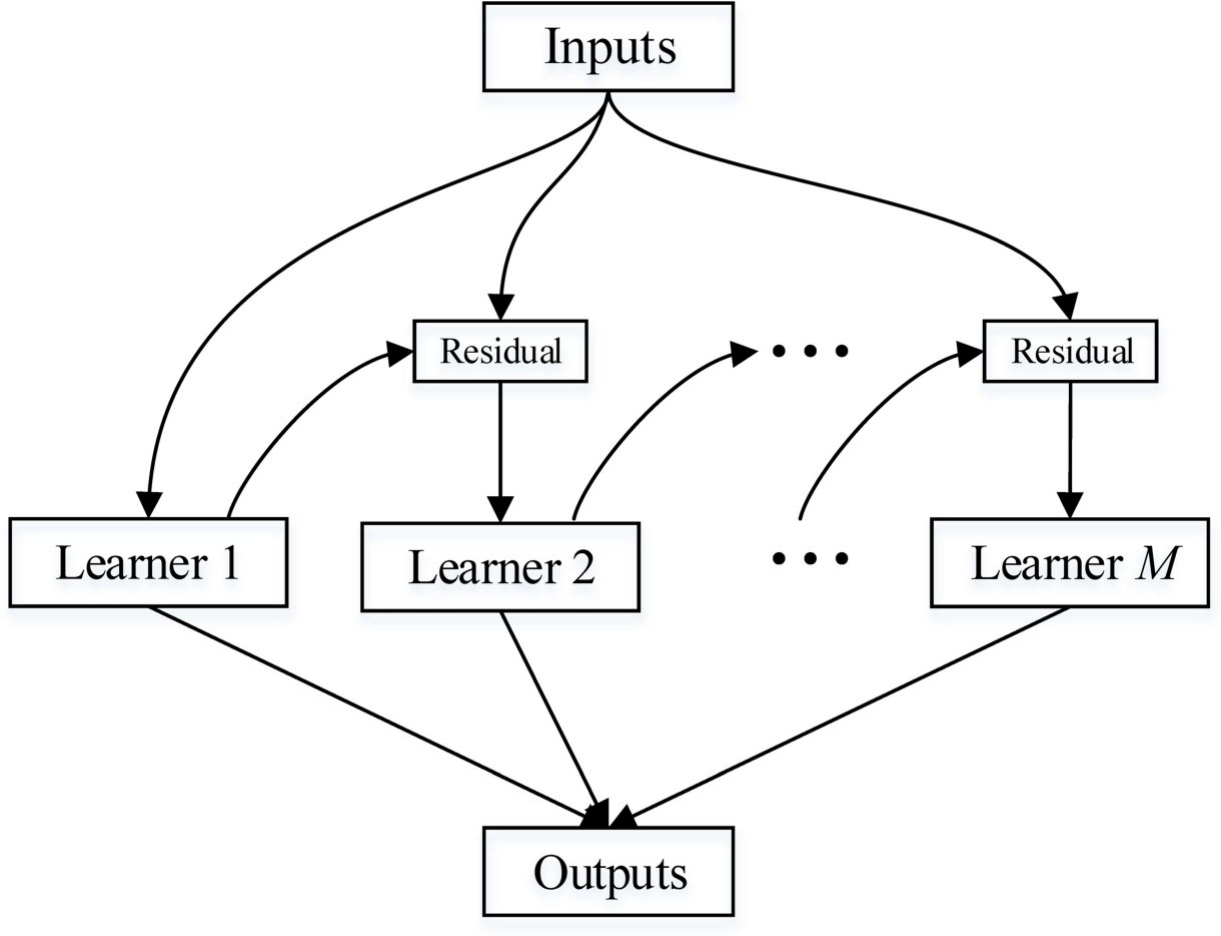
The architecture of GBDT. GBDT requires a total of *M* iterations. Each iteration generates a weak learner, and each learner is trained based on the residual of the previous learner. By using the gradient descent method, each iteration moves to the negative gradient direction of the loss function so that the loss function decreases, and the model is increasingly accurate.

A gradient boosting decision tree (GBDT) [10] was used to construct the model with Python-sklearn package. GBDT has been widely used in machine learning. It requires a total of *M* iterations. Each iteration generates a weak learner, and each learner is trained based on the residual of the previous learner. By using the gradient descent method, each iteration moves to the negative gradient direction of the loss function so that the loss function decreases, and the model is increasingly accurate. The architecture of the GBDT is shown in Fig 2.

#### The principle of GBDT is as follows

Samples in the training dataset were model inputs, and the predicted results were model outputs. Samples in the training dataset were expressed as {(*x*_1_, *y*_1_) … (*x*_*i*_, *y*_*i*_) … (*x*_*N*_, *y*_*N*_)}. The parameter *x* was an independent characteristic for diagnosing gastric cancer, and parameter *y* was a label (non-gastric cancer or gastric cancer). Sample (*x*_*i*_, *y*_*i*_) ranks *ith* in the training dataset. The total number of iterations was *M*. The principle was as follows.

1. First, we initialized the learner in the gradient boosting decision tree.
  $$f_{0}(x)=\arg\;\min_{\gamma }\sum_{i=1}^{N}L(y_{i},\gamma ),$$
  where *f*_*0*_*(x)* is the initialized learner, a tree with only one root node, γ is a constant value that minimizes the loss function, *N* is the number of training samples in the training dataset, *i* is a positive integer and 1≤ *i*≤ *N*.
  *L* is the loss function and defined as
  L(y,f(x))=log(1+exp(−yf(x))),
  where y∈{−1,+1} and *f*(*x*) is the output value of the training sample (*x*, *y*).
2. The total number of iterations was *M* in the GBDT. *f*_*m*_*(x)* is the learner obtained in iteration *m*, where *m* is a positive integer and 1≤ *m*≤ *M*. The strong learner *f*_*m-1*_*(x)* is obtained in the previous iteration (iteration *m*-1), and the loss function is *L*(*y*, *f*_*m-1*_*(x)*). The purpose of this iteration (iteration *m*) is to minimize the loss function.
  To update the learner in the GBDT. We calculated the negative gradient of the residual of the *ith* training sample in iteration *m*.
  $$r_{im}=-{\left[\frac{\partial L(y_{i},f(x_{i}))}{\partial f(x_{i})}\right]}_{f\left(x\right)=f_{m-1}\left(x\right)},$$
  where *r*_*im*_ is the negative gradient of the residual of the *ith* training sample in iteration *m*, and *f*(*x*_*i*_) is the output value of the training sample (*x*_*i*_, *y*_*i*_), that is, the predictive occurrence probability corresponding to the *ith* training sample.
  The *r*_*im*_ is used for the next iteration to fit a new regression tree *f*_*m*_(*x*). The corresponding leaf node region of *f*_*m*_(*x*) is *R*_*jm*_, where *j* is the number of leaf nodes in the leaf node region, *j is* a positive integer and 1≤ *j*≤ *J*.
3. Then, we calculated the best fit value for the leaf area.
  $$\gamma_{jm}=\arg\;\min_{\gamma }\sum_{x_{i}\in R_{jm}}L(y_{i},f_{m-1}(x_{i})+\gamma ),$$
  where γ_m_ is the best fit value for the *j* leaf node in iteration *m* and *f*_*m-*1_(*x*_*i*_) is the output value of the training sample (*x*_*i*_, *y*_*i*_), that is, the predictive occurrence probability corresponding to the *ith* training sample in iteration *m*-1.
4. Then, we updated the strong learner.
  $$f_{m}(x)=f_{m-1}(x)+\sum_{j=1}^{J}\gamma_{jm}I(x\in R_{jm}),$$
  where *I* is a unit vector.
5. Last, we generated strong learners.
  $$f(x)=f_{M}(x)=f_{0}(x)+\sum_{m=1}^{M}\sum_{j=1}^{J}\gamma_{jm}I(x\in R_{jm}),$$
  where *f*_*M*_*(x)* is the learner obtained in iteration *M*.

#### Tuning parameters of GBDT

The parameters of GBDT could be divided into boosting parameters and tree-specific parameters. Boosting parameters consisted of learning rate and number of trees. Tree-specific parameters consisted of min_samples_leaf and max_depth. We used k-fold cross-validation to tune the parameters. In our study, k = 5. The training dataset was split into a 5 folds where each fold was used as a validation dataset at some point. In the first iteration, the first fold was used to validate the model and the rest were used to train the model. In the second iteration, the second fold was used as the validation dataset while the rest served as the training dataset. This process was repeated until each fold of the 5 folds have been used as the validation dataset.

**Fig 3:**
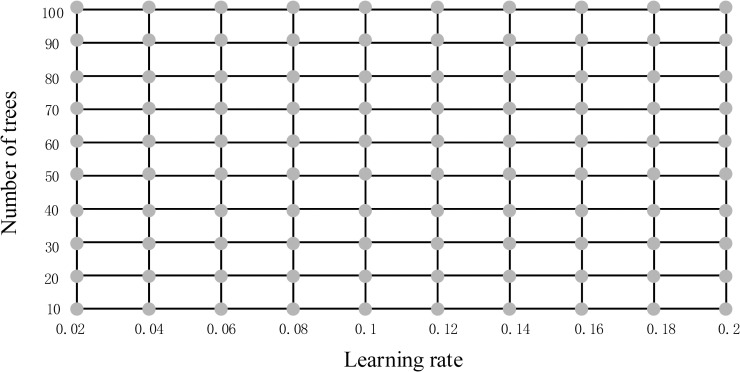
Grid search to select the optimum learning rate and number of trees. We chose the parameters on the grid and calculated the scores of AUC using the 5-fold cross-validation. After searching the entire grid, we selected the optimal parameters with highest score of AUC. The optimal parameters of learning rate and number of trees were 0.12 and 70, which won the highest score of 0.86.

Firstly, tree-specific parameters adopted default values of which min_samples_leaf = 1 and max_depth = 3, then we did a grid search to select the optimum learning rate and number of trees (Fig 3). The initial value of learning rate was 0.02 and the step was 0.02. The initial value of number of trees was 10 and the step was 10. We chose the parameters on the grid and calculated the scores of area under the receiver operating characteristic curve (AUC) using the 5-fold cross-validation. After searching the entire grid, we selected the optimal parameters with highest score of AUC. The optimal parameters of learning rate and number of trees were 0.12 and 70, which won the highest score of 0.86.

Secondly, the optimum learning rate and number of trees of 0.12 and 70 were used for determining the tree parameters. We set some initial values of tree-specific parameters: min_samples_leaf = 1 and max_depth = 3. Then we moved onto tuning the parameters detail by detail according to the result of 5-fold cross-validation to get more robust model. We have run 30 combinations and the ideal values were: min_samples_leaf = 10 and max_depth = 5.

### Testing algorithm

After constructing the diagnostic system, we used a test dataset consisting of 213 patients to evaluate the classification accuracy of the system. Receiver operating characteristic (ROC) curves were plotted by a set threshold. Sensitivity, specificity, positive predictive value, negative predictive value, and accuracy were calculated by standard definitions.

## Results

### Clinicopathologic characteristics

A total of 709 patients were enrolled in our study. A total of 398 patients were diagnosed with gastric cancer and 311 patients were non-gastric cancer (202 patients had benign gastric disease and 109 patients were health people). The clinical characteristics of the included patients are shown in Table 1. A total of 146 patients (36.7%) were female in the patients with gastric cancer, and 208 patients (66.9%) were female in the non-gastric cancer patients (*P*<0.05). Notably, patients with gastric cancer had significantly higher age, neutrophil count, NLR, RDW, Cr, Lpa, CEA, CA125, CA199 and CA724 than patients of non-gastric cancer (*P*<0.05 for all). Lymphocyte, Hb, Alb, ALT, TB, TG, HDL-C and LDL-C levels in patients with gastric cancer were significantly decreased (*P*<0.05 for all).

**Table 1:** Clinicopathologic characteristics of the subjects.

Characteristic	Non-gastric cancer (n = 311)	Gastric cancer (n = 398)	*P* value
Gender (M/F)	103/208	252/146	<0.01
Age(yr)	53(45–61)	66(57–72)	<0.01
Neutrophil count (10^9^/L)	3(2.5–3.7)	3.5(2.8–4.7)	<0.01
Lymphocyte count (10^9^/L)	1.8(1.4–2.2)	1.5(1.1–2)	<0.01
NLR	1.71(1.38–2.71)	2.34(1.7–3.58)	<0.01
Hb(g/L)	137(128–150)	127(106–141)	<0.01
RDW(%)	12.5(12.1–13)	13(12.3–14.1)	<0.01
Plt(10^9^/L)	214(181–254)	220(176–269)	0.214
Alb(g/L)	42.9(40.3–45.5)	38.2(34.25–41.9)	<0.01
ALT(U/L)	17(12–24)	14(11–20)	<0.01
TB(μmol/L)	12.5(9.8–16.1)	11.6(9–15.5)	0.031
Cr(μmol/L)	69.3(62.6–78.7)	76.9(66.4–88.0)	<0.01
TG (mmol/L)	1.205(0.92–1.78)	1.14(0.88–1.54)	0.017
HDL-C(mmol/L)	1.29(1.09–1.51)	1.10(0.89–1.28)	<0.01
LDL-C(mmol/L)	2.78(2.25–3.29)	2.58(2.02–3.10)	<0.01
Lpa(mg/L)	105(55–246)	153.5(79.5–299.3)	<0.01
CEA(μg/L)	1.6(1.1–2.5)	2.4(1.5–4.5)	<0.01
CA125(U/mL)	11.2(8.1–14.8)	12.45(8.6–21.2)	<0.01
CA199(U/mL)	9.65(6.7–14.4)	11.05(6.1–26.0)	<0.01
CA724(U/mL)	1.95(1.1–4.8)	2.4(1.2–7.3)	<0.01

Values are presented as medians (first and third quartiles) for continuous variables and absolute numbers for categorical data.

NLR, neutrophil lymphocyte ratio; Hb, hemoglobin; RDW, red cell distribution width; Hct, hematocrit; Plt, platelet; Alb, albumin; ALT, alanine transaminase; TB, total bilirubin; Cr, creatinine; TG, triglyceride; HDL-C, high-density lipoprotein cholesterol; LDL-C, low-density lipoprotein cholesterol; Lpa, lipoprotein; CEA, carcinoembryonic antigen; CA125, carbohydrate antigen 125; CA199, carbohydrate antigen 199; CA724, carbohydrate antigen 724.

Multivariate analysis further supported that gender, age, NLR, Hb, Alb, CEA, CA125 and CA199 were independent characteristics for diagnosing gastric cancer (Table 2).

**Table 2:** Multivariate analysis of independent characteristics associated with gastric cancer.

characteristics		OR	95% CI	*P* value	β
gender	female	Reference			
	male	3.2	2.16–4.74	<0.01	1.16
age(y)	<60	Reference			
	≥60	5.11	3.37–7.76	<0.01	1.63
NLR	<2	Reference			
	≥2	2.02	1.36–3.00	<0.01	0.7
Hb(mg/L)	<110	Reference			
	≥110	0.17	0.08–0.36	<0.01	-1.78
Alb(g/L)	<40	Reference			
	≥40	0.35	0.23–0.53	<0.01	-1.05
CEA(μg/L)	<5	Reference			
	≥5	2.9	1.34–6.27	<0.01	1.06
CA125(U/mL)	<35	Reference			
	≥35	6.72	2.21–2.4	<0.01	1.9
CA199(U/mL)	<37	Reference			
	≥37	2.88	1.23–6.77	0.015	1.06

OR, odds ratio; CI, confidence interval; β, beta coefficients; NLR, neutrophil lymphocyte ratio; Hb, hemoglobin; Alb, albumin; CEA, carcinoembryonic antigen; CA125, carbohydrate antigen 125; CA199, carbohydrate antigen 199.

The model was constructed according to the beta coefficients obtained for the independent characteristics. The clinical characteristics of the training and test dataset are shown in Table 3.

**Table 3:** Clinicopathologic characteristics of patients in the training and test dataset.

Characteristics		Training dataset (n = 496,%)		Test dataset (n = 213,%)
gender	Female		240(48.4%)	114(53.5%)
	Male		256(51.6%)	99(46.5%)
age(y)	<60		158(31.9%)	76(35.7%)
	≥60		338(68.1%)	137(64.3%)
NLR	<2		227(45.8%)	111(52.1%)
	≥2		269(54.2%)	102(47.9%)
Hb(mg/L)	<110		78(15.7%)	39(18.3%)
	≥110		418(84.3%)	174(81.7%)
Alb(g/L)	<40		223(45.0%)	93(43.7%)
	≥40		273(55.0%)	120(56.3%)
CEA(μg/L)	<5		415(83.7%)	191(89.7%)
	≥5		81(16.3%)	22(10.3%)
CA125(U/mL)	<35		442(89.1%)	196(92.0%)
	≥35		54(10.9%)	17(8.0%)
CA199(U/mL)	<37		438(88.3%)	188(88.3%)
	≥37		58(11.7%)	25(11.7%)
Gastric cancer or not	non-gastric cancer		212(42.7%)	99(46.5%)
	Gastric cancer		284(57.3%)	114(53.5%)

### Performance of machine learning

**Fig 4:**
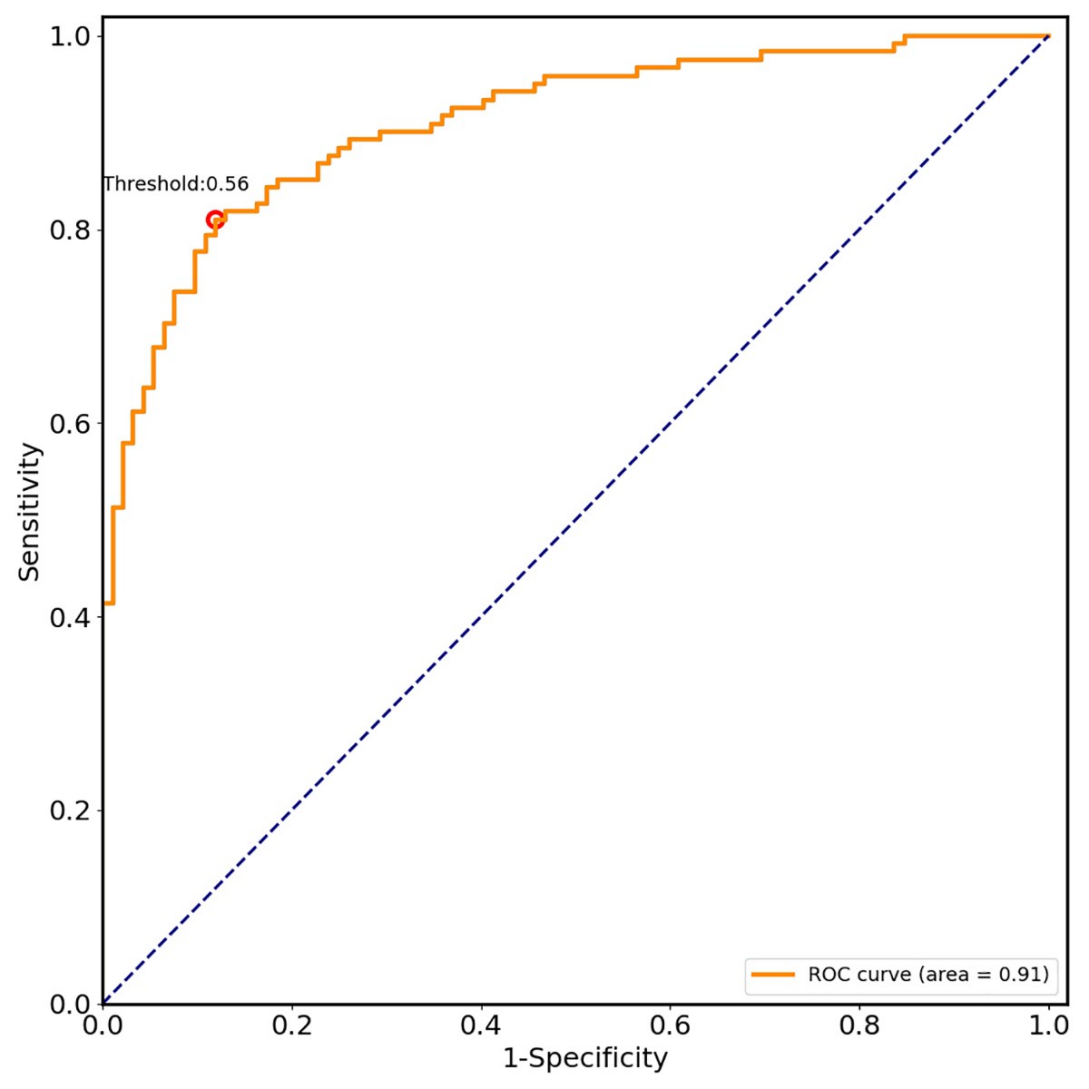
Receiver operating curve for the test dataset. According to GBDT, a predictive model was constructed to diagnose gastric cancer. We plotted a receiver operating curve of the probability of non-gastric cancer (negative) and gastric cancer (positive) classifications for the test dataset to assess the robustness of our model. The AUC was 91%.

According to GBDT, a predictive model was constructed to diagnose gastric cancer. We plotted a receiver operating curve of the probability of non-gastric cancer (negative) and gastric cancer (positive) classifications for the test dataset (Fig 4) to assess the robustness of our model. The area under the receiver operating characteristic curve (AUC) was 91%.

The performance of the model is shown in Table 4. The point for the optimal threshold appeared to be closest to the top-left of the plot, which maximized Yoden index (sensitivity+specificity–1). In our study, the optimal threshold was 0.56. At the threshold, sensitivity was 87.0% and specificity 84.1%. The overall accuracy of the model was 83.0%. Positive and negative predictive values were 83.0% and 87.8%, respectively.

**Table 4:** The performance of the model.

	accuracy, %	sensitivity, %	specificity, %	Positive predictive value, %	Negative predictive value, %
training dataset	85.9	88.0	83.4	87.0	84.7
test dataset	83.0	87.0	84.1	83.0	87.8

## Discussion

Early diagnosis and early treatment are crucial for improving the survival rate and reducing mortality [11]. Currently, the diagnosis of gastric cancer mainly depends on endoscopy. However, endoscopy is invasive, and some patients feel discomfort when doing it. In some rural areas, patients cannot afford expenses, which results in high mortality [5]. Therefore, it is of great significance to explore noninvasive characteristics or models to diagnose gastric cancer, which could reduce the medical cost and improve patient satisfaction.

A few researchers use genetics, proteomics and molecular biology to diagnose gastric cancer. For example, Zhou B et al. found the proteins identified by plasma proteomics could help distinguish EGC from healthy controls [12]. Wu J et al. stated that circulating microRNA-21 is a potential diagnostic biomarker in gastric cancer [13]. Watanabe Y et al. noted that several genes, such as adam23 and mint25, are methylated with higher frequency and therefore are analyzed as possible biomarkers [14]. Unfortunately, due to the restrictions of invasion, complexity and high cost, these achievements are not available in many hospitals and may impose a financial burden.

Tumor markers such as CEA, CA199, CA125, and CA724 are widely used clinically for the detection of gastric cancer. The reported sensitivity of CEA, CA199, CA125 or CA724 is approximately 40%, and combined detection of these characteristics can increase the sensitivity to 65% [15–17]. Sahin AG et al. found that the NLR is also associated with gastric cancer and that patients with gastric cancer have a significantly higher NLR [18]. Wu Y et al. showed that the combined use of the neutrophil-lymphocyte ratio, platelet-lymphocyte ratio and carcinoembryonic antigen could aid in the diagnosis of gastric cancer [19]. However, these available noninvasive characteristics are not satisfactory in their sensitivity and accuracy.

The recent development of machine learning offers the advantage of diagnosing gastric cancer accurately. Liu MM et al. applied data mining methods to predict gastric cancer, and the accuracy was 77% [20]. Su Y et al. diagnosed gastric cancer using decision tree classification of mass spectral data with an accuracy of 86.4% [21]. Some researchers use neural networks to diagnose gastric cancer in the area of endoscopic images with high sensitivity [8, 22].

Based on these studies, we combined the noninvasive characteristics with the help of machine learning to diagnose gastric cancer. In this study, we found that gender, age, NLR, Hb, Alb, CEA, CA125 and CA199 were independent characteristics for diagnosing gastric cancer. Gastric cancer was more common in older people and males. Hb and Alb levels in patients with gastric cancer were significantly decreased. Patients with gastric cancer had significantly higher NLR, CEA, CA125 and CA199 than patients of non-gastric cancer. These findings were consistent with previous reports [15–18, 23].

GBDT is widely used in machine learning. GBDT methods obtain good predictions when dealing with numerous factors and complicated relations among factors. It could make good use of weak classifiers for cascading and fully consider the weight of each classifier [10]. GBDT often works great with categorical and numerical values and is applicable to our study. We generated a GBDT model with high accuracy in distinguishing patients with gastric cancer from non-gastric cancer based on noninvasive characteristics. We tuned the parameters by k-fold cross-validation to deal with overfitting. Min_samples_leaf and max_depth were tuned to control over-fitting. Higher values of min_samples_leaf and lower max_depth prevented the model from learning relations which might be highly specific to the particular sample selected for a tree. To our knowledge, this was the first report of the use of GBDT to diagnose gastric cancer based on noninvasive characteristics. In addition, these characteristics are widely used clinically and inexpensive. Patients were initially screened by the model, and then the high-risk patients screened were confirmed by further endoscopy and pathology biopsy. Furthermore, the model obtained a high prediction performance. The model correctly predicted 83.0% in the test dataset, resulting in a positive predictive value of 83.0% and a negative predictive value of 87.8%.

This study had several limitations. First, the sample size was small, and all data were obtained from a single center. It was still far from sufficient to develop a reliable model. Further studies with many more cases and data from other centers are urgently required. Second, we only used GBDT to diagnose gastric cancer. Due to dependencies between weak learners, it was difficult to train data in parallel. In the next study, other related methods, such as neural networks and random forests, could also be used to construct the model.

In conclusion, we construct a GBDT model to diagnose gastric cancer with high sensitivity and accuracy, which is noninvasive and could reduce the medical cost. The model could be applied for auxiliary diagnosis of gastric cancer.

## Supporting information

S1 Checklist STROBE statement—checklist of items that should be included in reports of observational studies. (DOCX)

S1 Data (PDF)

## References

[1] Bray F, Ferlay J, Soerjomataram I, et al Global cancer statistics 2018: GLOBOCAN estimates of incidence and mortality worldwide for 36 cancers in 185 countries. *CA Cancer J Clin* 2018; 68:394–424. 10.3322/caac.21492 30207593

[2] Chen W, Zheng R, Baade PD, et al Cancer statistics in China, 2015. *CA Cancer J Clin* 2016; 66:115–132. 10.3322/caac.21338 26808342

[3] Zhang SW, Yang ZX, Zheng RS, et al Incidence and mortality of stomach cancer in China, 2013. *Zhonghua Zhong Liu Za Zhi* 2017; 39:547–552. 10.3760/cma.j.issn.0253-3766.2017.07.015 28728305

[4] Zheng RS, Sun KX, Zhang SW, et al Report of cancer epidemiology in China, 2015. *Zhonghua Zhong Liu Za Zhi* 2019; 41:19–28. 10.3760/cma.j.issn.0253-3766.2019.01.005 30678413

[5] Hou JZ, Dong SS, Yuan M, Zhong C. Patterns of death and life lost of gastric cancer in China cancer registration areas, 2013. Chinese Journal of Cancer Prevention and Treatment 2019; 26:986–990.

[6] Hornbrook MC, Goshen R, Choman E, et al Correction to: Early Colorectal Cancer Detected by Machine Learning Model Using Gender, Age, and Complete Blood Count Data. *Dig Dis Sci* 2017; 62:2719–2727. 10.1007/s10620-017-4722-8 28836087

[7] Das DK, Dutta PK. Efficient automated detection of mitotic cells from breast histological images using deep convolution neutral network with wavelet decomposed patches. *Comput Biol Med* 2019;104: 29–42. 10.1016/j.compbiomed.2018.11.001 30439598

[8] Zhu Y, Wang QC, Xu MD, et al Application of convolutional neural network in the diagnosis of the invasion depth of gastric cancer based on conventional endoscopy. *Gastrointest Endosc* 2019;89:806–815. 10.1016/j.gie.2018.11.011 30452913

[9] Yasar A, Saritas I, Korkmaz H. Computer-Aided Diagnosis System for Detection of Stomach Cancer with Image Processing Techniques. *J Med Syst* 2019; 43:99 10.1007/s10916-019-1203-y 30874907

[10] Friedman J H. Greedy function approximation: a gradient boosting machine. Annals of statistics 2001; 29: 1189–1232.

[11] Jun JK, Choi KS, Lee HY, et al Effectiveness of the Korean National Cancer Screening Program in Reducing Gastric Cancer Mortality. *Gastroenterology* 2017; 152:1319–1328. 10.1053/j.gastro.2017.01.029 28147224

[12] Zhou B, Zhou Z, Chen Y, et al Plasma proteomics-based identification of novel biomarkers in early gastric cancer. *Clin Biochem* 2020; 76:5–10. 10.1016/j.clinbiochem.2019.11.001 31765635

[13] Wu J, Li G, Wang Z, et al Circulating MicroRNA-21 Is a Potential Diagnostic Biomarker in Gastric Cancer. *Dis Markers* 2015; 2015: 435656 10.1155/2015/435656 26063956 PMC4433679

[14] Watanabe Y, Kim HS, Castoro RJ, et al Sensitive and Specific Detection of Early Gastric Cancer with DNA Methylation Analysis of Gastric Washes. *Gastroenterology* 2009;136:2149–58. 10.1053/j.gastro.2009.02.085 19375421 PMC2722957

[15] Zhu YB, Ge SH, Zhang LH, et al Clinical value of serum CEA, CA19-9, CA72-4 and CA242 in the diagnosis and prognosis of gastric cancer. *Zhonghua Wei Chang Wai Ke Za Zhi* 2012;15:161–4. 22368025

[16] Shitrit D, Zingerman B, Shitrit AB, Shlomi D, Kramer MR. Diagnostic Value of CYFRA 21–1, CEA, CA 19–9, CA 15–3, and CA 125 Assays in Pleural Effusions: Analysis of 116 Cases and Review of the Literature. *Oncologist* 2005; 10:501–7. 10.1634/theoncologist.10-7-501 16079317

[17] Liang Y, Wang W, Fang C, et al Clinical significance and diagnostic value of serum CEA, CA19-9 and CA72-4 in patients with gastric cancer. *Oncotarget* 2016; 7:49565–49573. 10.18632/oncotarget.10391 27385101 PMC5226529

[18] Sahin AG, Aydin C, Unver M, Pehlivanoglu K. Predictive Value of Preoperative Neutrophil Lymphocyte Ratio in Determining the Stage of Gastric Tumor. *Med Sci Monit* 2017;23:1973–1979. 10.12659/msm.900681 28437391 PMC5413293

[19] Wu Y, Jiang M, Qin Y, Lin F, Lai M. Single and combined use of neutrophil–lymphocyte ratio, platelet–lymphocyte ratio and carcinoembryonic antigen in diagnosing gastric cancer. *Clin Chim Acta* 2018;481:20–24. 10.1016/j.cca.2018.02.027 29476736

[20] Liu MM, Wen L, Liu YJ, et al Application of data mining methods to improve screening for the risk of early gastric cancer. *BMC Med Inform Decis Mak* 2018;18:121 10.1186/s12911-018-0689-4 30526601 PMC6284275

[21] Su Y, Shen J, Qian H, et al Diagnosis of gastric cancer using decision tree classification of mass spectral data. *Cancer Sci* 2007; 98:37–43. 10.1111/j.1349-7006.2006.00339.x 17052262 PMC11158238

[22] Hirasawa T, Aoyama K, Tanimoto T, et al Application of artificial intelligence using a convolutional neural network for detecting gastric cancer in endoscopic images. *Gastric Cancer* 2018; 21:653–660. 10.1007/s10120-018-0793-2 29335825

[23] Xue F, Lin F, Yin M, Feng N, et al Preoperative albumin/globulin ratio is a potential prognosis predicting biomarker in patients with resectable gastric cancer. *Turk J Gastroenterol* 2017; 28:439–445. 10.5152/tjg.2017.17167 29086711

