# Supplementary material for: Application of machine learning in the diagnosis of gastric cancer based on noninvasive characteristics
Source: PLoS One. 2020 Dec 31;15(12):e0244869. doi: 10.1371/journal.pone.0244869 (PMC7775073; doi:10.1371/journal.pone.0244869)
Supplement: S1 Checklist — (DOCX) [file pone.0244869.s001.docx]

STROBE Statement—checklist of items that should be included in reports of observational studies

|  | Item No. | Recommendation | Page  No. | Relevant text from manuscript |
| --- | --- | --- | --- | --- |
| **Title and abstract** | 1 | (*a*) Indicate the study’s design with a commonly used term in the title or the abstract | P2 | To construct a predictive model for the diagnosis of gastric cancer with high accuracy based on noninvasive characteristics. |
|  |  | (*b*) Provide in the abstract an informative and balanced summary of what was done and what was found | P3 | We construct a predictive model to diagnose gastric cancer with high sensitivity and specificity. The model is noninvasive and may reduce the medical cost. |
| Introduction | | | |  |
| Background/rationale | 2 | Explain the scientific background and rationale for the investigation being reported | P3 | Gastric cancer is a common malignancy with high incidence and mortality rates. Unfortunately, there are no [noninvasive](javascript:void(0);) characteristics defined for detecting gastric cancer with high sensitivity and specificity. Early diagnosis and early treatment are crucial for improving the survival rate and reducing the mortality of gastric cancer. |
| Objectives | 3 | State specific objectives, including any prespecified hypotheses | P4 | Therefore, we constructed a predictive model using machine learning to diagnose gastric cancer based on these noninvasive characteristics. |
| Methods | | | |  |
| Study design | 4 | Present key elements of study design early in the paper | P4-P6 | Fig 1. The procedure of data preparation and model construction. |
| Setting | 5 | Describe the setting, locations, and relevant dates, including periods of recruitment, exposure, follow-up, and data collection | P4 | We reviewed the medical records of 960 patients who were diagnosed with gastric cancer, benign gastric disease or health people from December 2018 to August 2019 at Zhejiang Provincial People's Hospital |
| Participants | 6 | (*a*) *Cohort study*—Give the eligibility criteria, and the sources and methods of selection of participants. Describe methods of follow-up  *Case-control study*—Give the eligibility criteria, and the sources and methods of case ascertainment and control selection. Give the rationale for the choice of cases and controls  *Cross-sectional study*—Give the eligibility criteria, and the sources and methods of selection of participants | P4 | The inclusion criteria were as follows: i) age >18 years, ii) histologically confirmed gastric cancer, benign gastric disease or health, iii) complete relevant data, iv) no other cancer. The exclusion criteria included the following: i) incomplete relevant data, ii) double cancers, and iii) recurrent gastric cancer. |
|  |  | (*b*) *Cohort study*—For matched studies, give matching criteria and number of exposed and unexposed  *Case-control study*—For matched studies, give matching criteria and the number of controls per case | P4、P12 | A total of 709 patients were enrolled in our study. A total of 398 patients were diagnosed with gastric cancer and 311 patients were non-gastric cancer (202 patients had benign gastric disease and 109 patients were health people). |
| Variables | 7 | Clearly define all outcomes, exposures, predictors, potential confounders, and effect modifiers. Give diagnostic criteria, if applicable | P5、P16 | Gastric cancer included adenocarcinoma, in situ carcinoma, malignant gastrointestinal stromal tumor, signet ring cell carcinoma, non-Hodgkin lymphoma, papillary carcinoma and tubular adenocarcinoma. Benign gastric disease included benign gastrointestinal stromal tumor, lipoma, leiomyoma, neurilemmoma, fibroxanthoma and gland polyps. Healthy people were defined as no obvious abnormalities in pathological examination.  We plotted a receiver operating curve of the probability of non-gastric cancer (negative) and gastric cancer (positive) classifications for the test dataset |
| Data sources/ measurement | 8* | For each variable of interest, give sources of data and details of methods of assessment (measurement). Describe comparability of assessment methods if there is more than one group |  |  |
| Bias | 9 | Describe any efforts to address potential sources of bias | N/A | N/A |
| Study size | 10 | Explain how the study size was arrived at | P5 | We learned that the sensitivity of the predictive model was 0.8 and the specificity was 0.75 based on preliminary data from our early observation of 80 subjects (40 of each group). We used PASS software (PASS, version 11.0) to estimate the sample size. It was found that at least 23 patients in each group were required, with a two-tailed test of α =0.05, 1 – β (the power) =0.90 and the ratio between groups 1:1 (prevalence rate 0.5). Considering a 10% loss of drop-out rate, at least 26 patients in each group were required. Thus the sample size of this study met with the requirement. |

Continued on next page

| Quantitative variables | 11 | Explain how quantitative variables were handled in the analyses. If applicable, describe which groupings were chosen and why | P6 | Measurement data were treated with the t-test if they followed the normal distribution or treated with the Mann-Whitney U test if they did not follow the normal distribution. |
| --- | --- | --- | --- | --- |
| Statistical methods | 12 | (*a*) Describe all statistical methods, including those used to control for confounding | P6-P7 | A univariate analysis was performed to evaluate the relationship between the noninvasive characteristics and diagnosis of gastric cancer. Measurement data were treated with the t-test if they followed the normal distribution or treated with the Mann-Whitney U test if they did not follow the normal distribution. Enumeration data were treated with the chi-square test. Significant characteristics were screened in the univariate analysis. Then, a multivariate analysis was performed using the significant characteristics to screen independent characteristics for diagnosing gastric cancer. Multivariable analysis was performed by logistic regression. A P-value of less than 0.05 was considered to be significant. Data were analyzed with SPSS software (SPSS, version 26.0, United States). |
|  |  | (*b*) Describe any methods used to examine subgroups and interactions | N/A | N/A |
|  |  | (*c*) Explain how missing data were addressed | P4-P5 | Notably, 32 patients with another cancer and 166 with recurrent gastric cancer were excluded; 53 patients who had insufficient data were also excluded. Finally, 709 patients were enrolled in our study to develop a predictive model. |
|  |  | (*d*) *Cohort study*—If applicable, explain how loss to follow-up was addressed  *Case-control study*—If applicable, explain how matching of cases and controls was addressed  *Cross-sectional study*—If applicable, describe analytical methods taking account of sampling strategy | P11 | A total of 709 patients were enrolled in our study. A total of 398 patients were diagnosed with gastric cancer and 311 patients were non-gastric cancer (202 patients had benign gastric disease and 109 patients were health people). |
|  |  | (*e*) Describe any sensitivity analyses | P11 | Receiver operating characteristic (ROC) curves were plotted by a set threshold. Sensitivity, specificity, positive predictive value, negative predictive value, and accuracy were calculated by standard definitions. |
| Results | | | | |
| Participants | 13* | (a) Report numbers of individuals at each stage of study—eg numbers potentially eligible, examined for eligibility, confirmed eligible, included in the study, completing follow-up, and analysed | P4 | Finally, 709 patients were enrolled in our study to develop a predictive model. |
|  |  | (b) Give reasons for non-participation at each stage | N/A | N/A |
|  |  | (c) Consider use of a flow diagram | P6 | Fig 1. The procedure of data preparation and model construction. |
| Descriptive data | 14* | (a) Give characteristics of study participants (eg demographic, clinical, social) and information on exposures and potential confounders | P12-P13 | Table 1. Clinicopathologic characteristics of the subjects. |
|  |  | (b) Indicate number of participants with missing data for each variable of interest | N/A | N/A |
|  |  | (c) *Cohort study*—Summarise follow-up time (eg, average and total amount) | N/A | N/A |
| Outcome data | 15* | *Cohort study*—Report numbers of outcome events or summary measures over time | N/A | N/A |
|  |  | *Case-control study—*Report numbers in each exposure category, or summary measures of exposure | P12-P13 | Table 1. Clinicopathologic characteristics of the subjects. |
|  |  | *Cross-sectional study—*Report numbers of outcome events or summary measures | N/A | N/A |
| Main results | 16 | (*a*) Give unadjusted estimates and, if applicable, confounder-adjusted estimates and their precision (eg, 95% confidence interval). Make clear which confounders were adjusted for and why they were included | P12-P14 | Table 2. Multivariate analysis of independent characteristics associated with gastric cancer. |
|  |  | (*b*) Report category boundaries when continuous variables were categorized | P12-P13 | Table 1. Clinicopathologic characteristics of the subjects. |
|  |  | (*c*) If relevant, consider translating estimates of relative risk into absolute risk for a meaningful time period | P13-P16 | Table 2. Multivariate analysis of independent characteristics associated with gastric cancer. |

Continued on next page

| Other analyses | 17 | Report other analyses done—eg analyses of subgroups and interactions, and sensitivity analyses | P17 | Table 4. The performance of the model. |
| --- | --- | --- | --- | --- |
| Discussion | | | | |
| Key results | 18 | Summarise key results with reference to study objectives | P17-P19 | Patients with gastric cancer had significantly higher NLR, CEA, CA125 and CA199 than patients of non-gastric cancer. These findings were consistent with previous reports [15-18, 24].  We generated a GBDT model with high accuracy in distinguishing patients with gastric cancer from non-gastric cancer based on noninvasive characteristics. |
| Limitations | 19 | Discuss limitations of the study, taking into account sources of potential bias or imprecision. Discuss both direction and magnitude of any potential bias | P19-P20 | This study had several limitations. First, the sample size was small, and all data were obtained from a single center. It was still far from sufficient to develop a reliable model, and we also did not have enough cases to validate the model. Further studies with many more cases and data from other centers are urgently required. Second, we only used GBDT to diagnose gastric cancer. Due to dependencies between weak learners, it was difficult to train data in parallel. In the next study, other related methods, such as neural networks and random forests, could also be used to construct the model. |
| Interpretation | 20 | Give a cautious overall interpretation of results considering objectives, limitations, multiplicity of analyses, results from similar studies, and other relevant evidence | P17-P20 | To our knowledge, this was the first report of the use of GBDT to diagnose gastric cancer based on noninvasive characteristics. In addition, these characteristics are widely used clinically and inexpensive. Patients were initially screened by the model, and then the high-risk patients screened were confirmed by further endoscopy and pathology biopsy. Furthermore, the model obtained a high prediction performance. The model correctly predicted 83.0% in the test dataset, resulting in a positive predictive value of 86.2% and a negative predictive value of 77.9%.  This study had several limitations. |
| Generalisability | 21 | Discuss the generalisability (external validity) of the study results | P20 | these characteristics are widely used clinically and inexpensive. Patients were initially screened by the model, and then the high-risk patients screened were confirmed by further endoscopy and pathology biopsy. Furthermore, the model obtained a high prediction performance. |
| Other information | |  | | |
| Funding | 22 | Give the source of funding and the role of the funders for the present study and, if applicable, for the original study on which the present article is based | N/A | This research was supported by grants from the Zhejiang Province Health Department  Fund (http://www.zjwjw.gov.cn/ ,No. 2018258924), the Key scientific and technology Project  of Zhejiang Province (http://kjt.zj.gov.cn/; No. 2019C03G2921039) .  The funders had no role in study design, data collection and analysis, decision to publish, or preparation of the manuscript |

*Give information separately for cases and controls in case-control studies and, if applicable, for exposed and unexposed groups in cohort and cross-sectional studies.

**Note:** An Explanation and Elaboration article discusses each checklist item and gives methodological background and published examples of transparent reporting. The STROBE checklist is best used in conjunction with this article (freely available on the Web sites of PLoS Medicine at http://www.plosmedicine.org/, Annals of Internal Medicine at http://www.annals.org/, and Epidemiology at http://www.epidem.com/). Information on the STROBE Initiative is available at www.strobe-statement.org.
