# Supplementary material for: Application of machine learning in the diagnosis of gastric cancer based on noninvasive characteristics
Source: PLoS One. 2020 Dec 31;15(12):e0244869. doi: 10.1371/journal.pone.0244869 (PMC7775073; doi:10.1371/journal.pone.0244869)
Supplement: S1 Data — (PDF) [file pone.0244869.s002.pdf]

label	gender	age	neu	lym		neu_lym	hb	hct	rdw	plt	alb
0	0	7	1.8	1	0	1.8	139	0.415	12.3	138	46.3
0	1	5	3.6	1.2	0	3	163	0.479	11.7	247	43.2
0	0	6	4.6	1.5	0	3.07	128	0.383	13.5	216	38.8
0	0	6	3	2	1	1.5	133	0.405	12.8	254	42
0	0	5	2.2	1.5	0	1.47	133	0.409	14.5	239	37.8
0	0	6	2.8	2.2	1	1.27	152	0.453	13.3	135	39.05
0	0	5	3.8	2	1	1.9	130	0.387	12.6	196	40.3
0	1	3	5.2	1.8	0	2.89	171	0.496	12.6	199	49.4
0	0	3	3.6	2.2	1	1.64	139	0.41	12	285	41.1
0	1	5	3.5	2	1	1.75	168	0.485	12.3	215	39.5
0	0	5	4	1.6	0	2.5	154	0.457	11.8	234	42.3
0	1	6	4.4	2.5	1	1.76	171	0.487	12.4	326	46.2
0	0	5	4	2.1	1	1.9	128	0.376	11.6	234	41.5
0	1	7	4.5	0.8	0	5.63	137	0.426	12.7	174	43.6
0	1	6	4.1	1.2	0	3.42	174	0.521	12	175	45.7
0	0	5	3.3	1.9	0	1.74	127	0.399	13.5	158	40.1
0	1	5	2.6	2	1	1.3	159	0.457	11.9	203	46.9
0	0	6	2.3	2.1	1	1.1	136	0.419	13	288	41.4
0	1	6	4.2	1.8	0	2.33	135	0.429	13.1	117	42.1
0	0	6	2.1	1.4	0	1.5	148	0.417	12.4	215	46.3
0	1	5	4.2	2.1	1	2	163	0.505	13	273	46.3
0	0	7	4.4	2.3	1	1.91	124	0.378	12.8	325	38.5
0	0	9	3.3	1.3	0	2.54	129	0.393	12.6	231	38.7
0	0	6	2.4	1.6	0	1.5	127	0.39	13.2	203	36.8
0	0	5	4.5	1.4	0	3.21	128	0.376	12.6	252	41
0	0	5	3.5	1	0	3.5	128	0.385	12.7	189	45.2
0	0	5	3.4	1.4	0	2.43	122	0.36	13.4	170	40.8
0	0	7	4	1.1	0	3.64	108	0.346	15.1	277	37.1
0	0	6	4.5	1.4	0	3.21	138	0.408	11.7	247	46
0	1	6	2.9	1.3	0	2.23	168	0.494	11.7	198	44.3
0	0	4	3.2	2.1	1	1.52	135	0.398	11.9	221	46.2
0	1	3	3.9	2	1	1.95	181	0.523	11.4	196	45.9
0	0	7	3.4	1.3	0	2.62	122	0.367	12.3	153	43.4
0	0	6	3	1.3	0	2.31	139	0.428	12.9	257	41.1
0	0	5	4.7	2	1	2.35	138	0.411	13.2	253	41.7
0	1	6	2.8	1.5	0	1.87	141	0.423	12.8	228	37.1
0	1	4	1.9	1.3	0	1.46	155	0.473	12.3	195	47.7
0	1	5	2.5	1.6	0	1.56	150	0.45	12.8	170	44.1
0	0	5	3.8	1.4	0	2.71	113	0.353	15.1	325	41
0	0	6	3	1.9	0	1.58	134	0.403	12.4	244	37.9
0	1	6	3.4	1.5	0	2.27	153	0.493	13.2	213	44
0	1	4	3.2	1.3	0	2.46	165	0.482	12.5	234	49.6
0	0	4	3.5	2.2	1	1.59	137	0.414	12.9	215	41.1
0	1	7	2.2	2.9	1	0.76	153	0.445	11.7	165	44.4
0	0	5	2.1	1.7	0	1.24	140	0.43	12.4	188	47.3
0	1	7	3.2	1.7	0	1.88	143	0.41	12.4	133	45.3
0	0	5	1.7	1.8	0	0.94	138	0.406	12.6	254	43.3
0	1	5	4.1	3	1	1.37	174	0.513	12.8	306	48.7
0	0	4	3.4	2.2	1	1.55	126	0.384	12.2	195	37.6
0	0	5	3.2	1.2	0	2.67	151	0.453	11.6	191	41.55
0	0	5	3.3	2.3	1	1.43	149	0.426	12.4	289	45.5
0	1	5	3.2	2.2	1	1.45	156	0.465	12.5	163	42.1
0	0	3	2.6	1.6	0	1.63	125	0.372	14.8	209	48.1

0	0	6	3.8	1.9	0	2	129	0.396	13	262	43.1
0	1	6	2.9	1.8	0	1.61	133	0.396	13	207	37.6
0	0	5	4	2	1	2	135	0.404	13.1	322	46.4
0	0	9	3	0.8	0	3.75	90	0.272	11.5	145	35.8
0	0	6	3.7	1.8	0	2.06	131	0.397	12.5	174	41
0	0	4	3.65	1.45	0	2.665	140.5	0.404	12.35	159	41.85
0	1	4	3.6	1.1	0	3.27	150	0.411	12.2	144	42.7
0	0	6	2.8	1.7	0	1.65	132	0.386	12.9	212	42.6
0	1	6	2.2	1.2	0	1.83	136	0.414	12.4	166	42.9
0	0	6	1.3	1.3	0	1	116	0.353	12.6	228	37.2
0	0	4	3.4	2.3	1	1.48	140	0.424	12.4	467	42.4
0	1	6	3.8	1	0	3.8	162	0.461	12.3	121	47.6
0	0	5	3	1.6	0	1.88	128	0.394	12.6	284	40.8
0	0	5	4.6	2.3	1	2	156	0.468	11.8	348	44.8
0	0	7	2.6	1.1	0	2.36	131	0.415	12.2	197	38.7
0	1	5	3.8	1.4	0	2.71	182	0.513	12.1	197	55.3
0	0	6	2.3	1.2	0	1.92	144	0.438	11.9	162	47
0	1	7	3.2	1.6	0	2	155	0.467	13.1	145	42.3
0	1	5	4.6	2.5	1	1.84	174	0.49	11.9	254	45.3
0	0	6	4.2	1.3	0	3.23	144	0.412	12.4	161	45.5
0	1	5	3.2	2.3	1	1.39	159	0.468	12.3	204	44.5
0	0	5	2.1	1.5	0	1.4	151	0.47	12.4	269	47.5
0	0	6	4.3	2.3	1	1.87	153	0.472	12.9	249	47.2
0	1	7	3	2.6	1	1.15	153	0.449	12.8	114	38.1
0	0	6	1.9	1.8	0	1.06	139	0.426	12.3	240	45.5
0	0	7	1.7	1.3	0	1.31	131	0.393	13.3	186	44.8
0	0	6	2.8	1.5	0	1.87	143	0.428	11.8	197	50.8
0	0	5	2.7	2.1	1	1.29	124	0.378	12.4	281	38.8
0	1	4	3	3.2	1	0.94	169	0.501	11.6	196	50
0	0	7	2.3	1.9	0	1.21	125	0.386	11.6	245	39
0	1	4	2.3	1.9	0	1.21	168	0.475	12	204	43.2
0	0	5	3.4	2.1	1	1.62	125	0.381	11.9	216	39.3
0	1	6	2.8	2.2	1	1.27	150	0.465	13.4	167	42.5
0	0	4	2.9	1.3	0	2.23	122	0.367	12.5	218	44.2
0	0	4	2.3	1.3	0	1.77	124	0.381	13.3	180	46.6
0	1	5	5.7	1.3	0	4.38	145	0.429	11.9	147	41.85
0	1	5	2.1	1	0	2.1	144	0.445	11.7	97	37.1
0	0	8	1.8	1.4	0	1.29	125	0.367	12.8	241	36.4
0	0	3	2.6	1.6	0	1.63	122	0.372	12.4	223	39
0	1	7	5.2	1.8	0	2.89	150	0.453	12.8	210	39.8
0	1	5	3.7	3.1	1	1.19	144	0.429	13.3	216	38.1
0	0	7	2.6	3.2	1	0.81	124	0.377	12.1	246	14.2
0	0	3	3.5	2	1	1.75	134	0.392	12.5	342	44.3
0	0	6	1.9	2.6	1	0.73	134	0.423	12.9	143	36.3
0	1	4	5.9	2.2	1	2.68	168	0.505	12.6	224	49.7
0	0	5	2.5	2.3	1	1.09	138	0.411	12.5	291	44.6
0	1	4	4.7	2.3	1	2.04	172	0.502	12.5	267	48.3
0	1	3	3.1	2.4	1	1.29	175	0.503	13	268	48.8
0	1	3	3.9	3.1	1	1.26	177	0.519	12.9	155	49.2
0	1	3	3.6	2.4	1	1.5	162	0.477	12.7	187	48
0	1	7	4.9	2.4	1	2.04	158	0.468	12.9	252	50.1
0	1	5	3.8	1.7	0	2.24	142	0.417	12.9	211	43.9
0	0	6	5	2.4	1	2.08	136	0.408	11.8	251	37.7
0	0	4	3.3	1.5	0	2.2	139	0.421	12.5	219	42.9

0	1	4	3	1	0	3	36	0.415	12.7	202	40.7
0	1	4	6.2	4.1	1	1.51	177	0.524	12.1	314	48.4
0	0	4	2	1.2	0	1.67	109	0.346	15.5	300	43.5
0	0	6	2.7	1.8	0	1.5	128	0.382	12.8	218	40.1
0	1	6	2	1.2	0	1.67	148	0.432	13.1	200	35.8
0	0	5	3.2	2	1	1.6	135	0.407	12.9	270	36.9
0	1	6	2	1.7	0	1.18	141	0.42	11.6	139	42.3
0	0	5	2.6	1.9	0	1.37	136	0.412	13.1	124	42.7
0	0	4	4	2	1	2	146	0.437	11.6	262	42.9
0	1	4	3.9	2	1	1.95	169	0.485	12.6	256	47.3
0	0	7	3.1	1.3	0	2.38	144	0.425	11.5	295	49.8
0	0	9	4.5	1.3	0	3.46	129	0.39	13.1	156	45.9
0	0	6	2.8	2.2	1	1.27	136	0.394	11.9	187	42
0	0	7	2.4	1	0	2.4	157	0.48	12.1	214	52.4
0	0	4	2.4	1.1	0	2.18	111	0.35	17	196	47.2
0	0	5	2.3	1.8	0	1.28	136	0.411	12.3	284	41.4
0	1	4	3.7	1.6	0	2.31	169	0.479	12	208	40.6
0	0	5	4.9	1.8	0	2.72	131	0.377	12.2	198	38.8
0	0	5	2	1.4	0	1.43	130	0.39	12	200	44.6
0	0	5	2.8	1.1	0	2.55	130	0.401	13.2	174	47.2
0	0	6	0.9	4.2	1	0.21	117	0.34	13.7	236	31.5
0	0	3	2.1	2.2	1	0.95	126	0.373	11.3	269	41
0	1	2	4	3.5	1	1.14	157	0.463	12.2	272	41.8
0	0	7	3.4	1.6	0	2.13	131	0.405	12.6	190	44.6
0	0	5	2.7	1.1	0	2.45	135	0.407	12	183	35.7
0	1	5	2.6	1.5	0	1.73	149	0.447	12.4	234	48.8
0	1	5	3.5	1.7	0	2.06	152	0.444	12.6	241	41.9
0	0	5	1.4	1	0	1.4	123	0.382	12.3	189	37.2
0	0	6	2.5	1.9	0	1.32	124	0.378	11.7	270	39.5
0	0	7	3.7	1.9	0	1.95	128	0.382	12	296	39.9
0	0	5	2.6	1.5	0	1.73	150	0.451	13.1	268	42.3
0	1	4	3.6	1.5	0	2.4	156	0.428	11.9	307	44.6
0	0	4	2.1	1.6	0	1.31	126	0.388	12.1	267	41.4
0	0	7	4.1	1.8	0	2.28	129	0.385	12.1	250	44.3
0	0	5	6.6	0.8	0	8.25	111	0.354	17	248	41.5
0	1	5	3	1.3	0	2.31	165	0.48	11.1	224	45.1
0	1	3	5.5	0.5	0	11	145	0.434	12.1	200	45.9
0	0	7	3.9	2.3	1	1.7	136	0.393	12.7	346	42
0	0	5	2.9	1.9	0	1.53	137	0.401	13.8	144	46.8
0	0	7	2.3	1	0	2.3	135	0.389	11.6	145	38.1
0	0	4	3.6	1.9	0	1.89	125	0.385	12.3	284	37.4
0	0	5	2.7	2.4	1	1.13	138	0.422	12	181	39.7
0	1	5	2.7	1.2	0	2.25	150	0.456	12.5	137	39.4
0	1	5	5.6	1.9	0	2.95	138	0.425	13.4	178	36.2
0	0	5	2.6	1.7	0	1.53	132	0.411	11.8	213	39
0	0	5	2.4	1.6	0	1.5	126	0.397	12.9	124	38.9
0	1	5	5	1.8	0	2.78	171	0.507	12.9	222	47.6
0	0	5	2.6	0.9	0	2.89	127	0.387	12.5	225	51.3
0	1	5	3.6	1.4	0	2.57	142	0.43	13	208	40.8
0	1	5	3.4	2.4	1	1.42	150	0.444	12.3	268	39.8
0	0	6	3.4	2	1	1.7	142	0.423	12.2	269	44.1
0	0	5	1.9	2.2	1	0.86	131	0.398	11.1	196	43
0	0	5	4.8	2	1	2.4	143	0.43	11.9	269	45.9
0	0	6	2.1	2.1	1	1	140	0.426	13.3	199	41.5

0	1	5	3.7	2	1	1.85	153	0.448	11.8	232	41.6
0	1	5	2.5	1.6	0	1.56	154	0.451	12.4	198	41.8
0	0	7	2.8	2.3	1	1.22	117	0.336	11.5	143	38.4
0	0	4	2.7	1.7	0	1.59	139	0.415	12.4	219	45.6
0	0	5	4.9	1.9	0	2.58	138	0.413	12.7	274	47.4
0	0	7	2.2	1.2	0	1.83	119	0.352	12.5	209	42
0	1	2	3.2	1.7	0	1.88	175	0.49	11.6	243	50.5
0	0	6	3.4	2.1	1	1.62	128	0.405	12	236	39.4
0	0	7	3.4	0.7	0	4.86	76	0.264	17.4	273	43.1
0	0	7	1.8	0.5	0	3.6	143	0.421	12.2	91	37.8
0	1	4	4.9	4.8	1	1.02	152	0.464	13.3	209	44.8
0	0	3	5	1.9	0	2.63	137	0.408	13	237	44
0	0	7	3	2.8	1	1.07	123	0.372	12	211	42.7
0	0	7	3.2	0.8	0	4	121	0.373	12.4	157	44.1
0	0	6	3.2	1.6	0	2	132	0.379	11.8	297	52.1
0	0	5	3.5	2.2	1	1.59	109	0.309	12.7	281	40.5
0	0	6	2	1.8	0	1.11	134	0.395	12.4	187	44.8
0	0	6	2.9	2	1	1.45	138	0.415	13.2	193	47.7
0	0	7	2.3	1.7	0	1.35	128	0.383	12.8	336	43
0	0	5	2.6	1.9	0	1.37	79	0.28	17.5	335	45
0	1	4	4.1	2.4	1	1.71	175	0.496	12.7	320	49.5
0	0	7	7.3	1	0	7.3	123	0.371	13.1	163	43.2
0	0	5	2.9	1	0	2.9	139	0.394	11.9	191	44.3
0	1	7	2.1	1.4	0	1.5	152	0.454	13	148	44.8
0	1	4	4.6	2.8	1	1.64	161	0.478	12.4	288	41.4
0	0	4	4.2	1.4	0	3	110	0.341	13.2	197	48.9
0	0	5	3	2.2	1	1.36	136	0.4	13	229	39.8
0	0	6	1.4	3	1	0.47	138	0.41	12.7	192	43.3
0	0	6	4.1	1.5	0	2.73	124	0.381	13.2	130	43
0	1	6	1.7	0.8	0	2.13	136	0.413	12.9	89	42.2
0	0	5	2.5	2.1	1	1.19	134	0.399	12.3	219	45.2
0	1	3	3.2	1.9	0	1.68	160	0.456	12	248	47.5
0	1	6	5.5	2.7	1	2.04	166	0.512	13.3	247	43.1
0	0	5	3.4	2.2	1	1.55	139	0.403	11.8	258	45.8
0	1	3	2.5	1.8	0	1.39	146	0.473	15.8	279	49.2
0	0	4	2.5	1.1	0	2.27	118	0.352	13.2	128	39.4
0	1	4	2.3	1.2	0	1.92	156	0.41	11.7	140	42.6
0	0	5	2.5	1.1	0	2.27	81	0.282	17.3	410	39.6
0	0	4	4	1.3	0	3.08	117	0.354	12.2	141	46.7
0	0	4	3.2	2.7	1	1.19	135	0.398	12.9	349	43
0	0	7	2.2	2.2	1	1	121	0.36	13.3	188	36.1
0	0	7	3.1	1.2	0	2.58	133	0.382	12.4	181	41.2
0	0	6	3.1	1.4	0	2.21	138	0.393	13	274	46.6
0	1	6	2	1.8	0	1.11	166	0.485	13.1	196	44.9
0	0	4	3.3	1.1	0	3	91	0.295	16.2	270	40.4
0	0	5	1.8	2.2	1	0.82	149	0.424	11.2	181	48.9
0	1	5	3	2.2	1	1.36	161	0.463	12.3	248	46.2
0	0	6	2.6	1.4	0	1.86	131	0.386	13.1	174	44.2
0	0	6	3.2	2.4	1	1.33	129	0.386	87.7	176	42.5
0	1	5	4.3	2.8	1	1.54	156	0.437	11.9	327	41.5
0	0	6	3.2	1.9	0	1.68	142	0.433	13.3	214	48.7
0	0	6	3.9	2.5	1	1.56	146	0.426	12.2	269	41
0	1	4	3.1	2	1	1.55	159	0.461	12.4	202	45.3
0	0	5	2.6	2.1	1	1.24	134	0.399	12.7	177	43.7

0	0	5	3.6	2.6	1	1.38	131	0.378	12.6	290	36.2
0	1	7	2.6	1.8	0	1.44	139	0.402	11.8	175	43.8
0	0	6	2.3	1.9	0	1.21	140	0.412	13.1	240	41.6
0	0	5	2.7	2.3	1	1.17	125	0.381	13.7	200	46.6
0	0	6	2.6	2.4	1	1.08	142	0.42	11.7	233	44.8
0	0	6	3.2	2.9	1	1.1	123	0.376	12.8	234	39.5
0	1	2	2.3	2.6	1	0.88	165	0.495	11.7	213	49.1
0	0	5	3	1.4	0	2.14	128	0.392	11.9	370	40.9
0	0	6	3.1	1.4	0	2.21	130	0.392	13.8	220	39.8
0	1	5	2.7	1.1	0	2.45	143	0.426	12.7	194	45.2
0	0	7	2.8	2.3	1	1.22	130	0.39	12.8	169	41.1
0	1	4	4.4	2	1	2.2	154	0.474	13.2	159	41.7
0	0	7	2.4	2.4	1	1	144	0.426	12.5	220	42.3
0	0	5	2.5	1.9	0	1.32	136	0.395	12.5	236	44.8
0	0	4	3.1	2.5	1	1.24	126	0.382	14.9	320	44.6
0	1	6	2.3	2	1	1.15	136	0.403	12.6	250	42.4
0	0	7	3.7	1.6	0	2.31	132	0.383	14.6	196	41.5
0	0	6	2.7	1.8	0	1.5	137	0.397	11.9	231	44.2
0	0	6	2.5	1.5	0	1.67	118	0.362	13.5	102	37.3
0	0	6	2.3	1.4	0	1.64	115	0.367	13.1	196	41.6
0	1	5	3.5	4	1	0.88	165	0.488	13	146	44.6
0	0	6	2.4	1.5	0	1.6	142	0.422	12.8	179	43.8
0	0	4	3.5	1.5	0	2.33	134	0.381	11.6	185	47.4
0	0	6	4.4	1.1	0	4	129	0.387	12.6	137	47.2
0	0	5	2.2	2	1	1.1	113	0.339	12.5	231	41.1
0	0	8	2.6	1.2	0	2.17	112	0.327	12.6	212	48.6
0	0	4	2.6	1.7	0	1.53	127	0.378	11.2	282	39.8
0	0	6	3.2	1	0	3.2	126	0.39	12.5	51	40.3
0	0	2	2.6	1.6	0	1.63	135	0.39	12.4	265	47.1
0	0	7	2.3	1.7	0	1.35	124	0.374	13.6	100	43.6
0	0	6	3.9	2.4	1	1.63	144	0.423	11.9	270	47.7
0	0	6	2.9	1.8	0	1.61	136	0.408	12.3	202	45
0	1	6	3.2	1.3	0	2.46	141	0.415	12.6	175	43.6
0	0	6	2.4	1.3	0	1.85	1222	0.368	12.7	186	39.9
0	0	4	2.9	2	1	1.45	114	0.359	14.7	274	41.3
0	0	6	2.9	2.4	1	1.21	137	0.411	12.4	202	40.4
0	1	6	3.4	1.5	0	2.27	137	0.412	13.5	225	40
0	1	5	2.6	3.1	1	0.84	135	0.404	12.7	214	38.5
0	0	6	3.2	1.9	0	1.68	140	0.417	12.4	181	43.7
0	0	6	2.6	2.1	1	1.24	131	0.39	12.1	303	40.9
0	0	5	3.7	2.5	1	1.48	134	0.404	12.4	294	46
0	1	6	2.3	1.5	0	1.53	150	0.44	12	201	47.3
0	1	5	4.4	2	1	2.2	149	0.432	12.3	238	42.6
0	0	4	2.6	1.7	0	1.53	129	0.387	13.5	235	46.9
0	1	6	3.3	2.1	1	1.57	135	0.412	12	210	42.2
0	0	4	2	0.7	0	2.86	128	0.394	14	212	41.4
0	1	4	2.6	1.7	0	1.53	161	0.48	12.2	259	45.9
0	0	6	2.5	2.5	1	1	128	0.39	12.6	282	40
0	1	6	3.9	2.1	1	1.86	140	0.402	11.9	246	45.1
0	0	4	2.5	1.5	0	1.67	124	0.359	12.3	186	40.4
0	0	6	2.2	1.6	0	1.38	146	0.423	12.6	196	38.2
0	0	5	2.7	0.9	0	3	79	0.27	17.2	249	36
0	1	6	3.4	1.4	0	2.43	153	0.447	13.2	309	45.8
0	1	5	2.9	0.9	0	3.22	152	0.446	12.8	184	45.4

0	1	6	2.2	1.4	0	1.57	140	0.408	12.6	147	40.5
0	0	6	3.5	1.9	0	1.84	130	0.392	11.9	253	42.8
0	0	5	4.1	1.2	0	3.42	143	0.418	12.3	197	42.7
0	1	3	2.8	2	1	1.4	154	0.461	11.9	149	47.3
0	1	7	2.6	1.1	0	2.36	119	0.357	13.4	171	32
0	0	5	2.1	1.2	0	1.75	131	0.387	11.8	225	38.3
0	0	4	2.1	1.7	0	1.24	114	0.352	13.9	260	43.4
0	0	3	3.6	1.6	0	2.25	163	0.468	12.7	176	45.5
0	0	5	3.2	0.9	0	3.56	128	0.393	12.8	295	44.4
0	0	3	4.1	3.5	1	1.17	150	0.45	11.9	186	44.7
0	0	6	2.2	1.2	0	1.83	121	0.37	12	191	39.9
0	1	4	2.8	1.9	0	1.47	157	0.45	11.9	167	45.2
0	1	6	4	0.7	0	5.71	155	0.444	11.8	178	48.7
0	1	6	2.4	1.7	0	1.41	152	0.456	12.4	190	35.3
0	0	6	2.3	1.6	0	1.44	119	0.354	12.5	284	44.5
0	0	6	2	2.2	1	0.91	106	0.344	15.7	235	40.3
0	0	5	4.1	2.8	1	1.46	133	0.391	11.9	188	36.1
0	0	4	2.3	1.5	0	1.53	112	0.347	13.8	222	39.1
0	0	6	3.2	0.8	0	4	144	0.431	13.2	176	44
0	0	5	3	2.1	1	1.43	133	0.396	12.3	272	39.7
0	0	4	4.1	2.5	1	1.64	131	0.403	13.4	329	38.5
0	0	6	3.2	1.5	0	2.13	134	0.413	11.8	177	43.5
0	1	3	3	2	1	1.5	150	0.424	12	161	42.2
0	0	7	2.9	1.6	0	1.81	131	0.392	12.2	149	41.7
0	0	7	3.8	2.8	1	1.36	136	0.406	12.9	235	44.6
0	0	3	3.9	1.7	0	2.29	127	0.376	12.5	179	47.4
0	0	5	2.6	1.3	0	2	135	0.414	12.7	220	39.9
0	0	4	4.4	1.9	0	2.32	123	0.38	12.2	224	34.8
0	0	5	2.3	1.6	0	1.44	78	0.267	19.4	351	40
0	0	6	2.8	2.9	1	0.97	116	0.351	12.6	256	36.8
0	1	6	2.5	1.3	0	1.92	144	0.429	12.6	158	33.8
0	0	5	3.9	1.4	0	2.79	137	0.393	11.7	289	48.8
0	0	7	3	1.6	0	1.88	144	0.446	12.4	193	44.6
0	0	5	3.6	1	0	3.6	146	0.431	13	223	43
0	0	6	2.9	2.4	1	1.21	118	0.349	12.1	225	41.4
0	1	6	2.3	1.3	0	1.77	145	0.407	11.9	153	39.2
0	1	6	1.9	1.8	0	1.06	133	0.394	12.3	149	44.5
0	0	5	3.6	1.7	0	2.12	130	0.367	11.9	149	42.7
0	1	7	4.1	3.1	1	1.32	157	0.467	13	279	46.2
0	0	4	3.4	1.2	0	2.83	118	0.378	13	130	47.2
0	0	7	3.1	1.3	0	2.38	144	0.433	12.4	180	43.7
0	0	7	3.8	2.2	1	1.73	127	0.389	12.9	246	45
1	0	6	3.4	1	0	3.4	70	0.218	15.5	299	26.6
1	1	7	4	1	0	4	105	0.33	13.8	296	23.6
1	1	7	4.4	2	1	2.2	133	0.4	12	236	37
1	0	7	1.8	1.3	0	1.38	119	0.352	13.5	117	21.3
1	0	5	2.6	1.8	0	1.44	124	0.372	13	208	40.4
1	1	7	2.1	1.2	0	1.75	145	0.421	13	216	43.4
1	1	6	5.8	2	1	2.9	130	0.387	13.2	235	37.7
1	1	6	2	2.1	1	0.95	113	0.346	13.4	136	33
1	0	5	1.8	2.3	1	0.78	87	0.295	19.2	326	36.7
1	0	5	1.6	1.6	0	1	101	0.334	15.4	154	37.3
1	1	7	7	1.6	0	4.38	122	0.353	12.5	222	35.8
1	0	6	3	1.7	0	1.76	130	0.397	13	277	36.9

1	0	7	3.5	1.9	0	1.84	148	0.44	12.2	234	45.3
1	1	6	4.1	2.2	1	1.86	141	0.438	12.7	212	42.1
1	0	6	6.5	2	1	3.25	144	0.453	12.2	245	41.7
1	0	5	5	1.1	0	4.55	129	0.384	14	224	42.1
1	1	8	4.9	1.9	0	2.58	140	0.409	12.2	203	32.1
1	1	7	2.3	1.6	0	1.44	101	0.302	13.7	265	37.4
1	1	7	4	1.7	0	2.35	160	0.475	13	180	36.2
1	1	6	3.4	2.3	1	1.48	139	0.421	13.5	294	38.3
1	0	6	3.1	1.2	0	2.58	91	0.268	13.1	192	30.5
1	0	7	2.8	1.8	0	1.56	130	0.4	14.5	256	39.7
1	0	8	1.9	1.1	0	1.73	80	0.256	14.8	172	32.4
1	1	6	3.3	1.4	0	2.36	139	0.416	12.6	161	40
1	1	6	4	1.8	0	2.22	145	0.436	12.6	232	46.3
1	0	5	2.5	0.8	0	3.13	110	0.342	11.9	131	40.6
1	0	6	3.5	1.4	0	2.5	125	0.377	12.8	209	42.5
1	1	5	3.2	1	0	3.2	81	0.258	15	103	28.6
1	0	7	4.9	0.7	0	7	95	0.293	14.6	300	35.5
1	1	9	4.4	0.4	0	11	92	0.287	14.6	155	26.6
1	0	7	4.9	2.5	1	1.96	148	0.446	13	264	46.5
1	0	5	5.6	2.7	1	2.07	138	0.408	12.1	188	43
1	1	6	3.1	2.4	1	1.29	143	0.404	11.8	228	14.8
1	1	5	4.2	2	1	2.1	143	0.434	13.3	140	37.1
1	1	8	3.2	0.9	0	3.56	61	0.204	17.1	157	23.3
1	1	7	2.6	1.7	0	1.53	127	0.391	13.3	400	33.8
1	1	8	3	1.1	0	2.73	142	0.427	12	156	32.3
1	1	7	3.3	1.4	0	2.36	101	0.305	12.7	251	35.5
1	1	7	2.7	1.1	0	2.45	161	0.458	12	200	39.7
1	1	6	3.3	1.1	0	3	54	0.183	19.8	375	31.5
1	0	6	2.2	1.3	0	1.69	111	0.349	12.8	202	39.4
1	1	8	2.8	1.9	0	1.47	150	0.438	12.7	174	36.9
1	1	8	2.8	1.2	0	2.33	160	0.478	13.2	265	40.2
1	1	8	3.7	1.3	0	2.85	131	0.392	12.7	204	38.9
1	0	5	2.3	1.2	0	1.92	154	0.463	12.4	202	46.8
1	0	5	3.8	0.8	0	4.75	128	0.388	12.7	250	41.3
1	0	6	2.8	0.9	0	3.11	101	0.324	14.4	374	42
1	0	5	3.2	2	1	1.6	145	0.43	11.9	267	44.9
1	1	6	4.8	2	1	2.4	154	0.458	13	232	43.3
1	0	7	7.2	0.8	0	9	56	0.191	14.8	320	31.8
1	0	7	2.2	1.9	0	1.16	133	0.401	13	299	37.7
1	1	6	3.2	0.8	0	4	144	0.43	12	142	44.3
1	0	4	3.5	2	1	1.75	137	0.385	12.6	238	14.3
1	1	6	3.4	3.2	1	1.06	99	0.329	19.9	444	40.3
1	1	6	3.1	2	1	1.55	152	0.441	12.8	148	35.3
1	1	8	4.2	1.5	0	2.8	133	0.399	13.4	269	37.3
1	1	7	3.2	1.1	0	2.91	126	0.381	14.7	162	33.4
1	0	5	2.9	1.4	0	2.07	106	0.338	13.6	441	44.1
1	1	7	3.8	2	1	1.9	155	0.463	12.5	167	39.9
1	0	8	2.8	1.3	0	2.15	102	0.325	13.9	286	38.2
1	0	6	2	2.1	1	0.95	120	0.376	12.3	235	36.9
1	1	9	3.5	1.9	0	1.84	97	0.344	27.1	186	38.7
1	1	9	4.6	1.7	0	2.71	103	0.326	13.2	207	29.2
1	0	8	4.9	2.4	1	2.04	124	0.335	12.7	186	36.4
1	1	7	5.6	2.2	1	2.55	120	0.37	12.6	222	33.3
1	0	7	8.3	2.3	1	3.61	67	0.239	21.1	447	34.7

1	1	7	4.5	2.5	1	1.8	114	0.364	14.2	409	38.2
1	1	8	5.4	0.8	0	6.75	75	0.25	26.9	296	29.8
1	0	6	2.6	1.2	0	2.17	129	0.389	11.8	270	13
1	1	7	2.4	1.8	0	1.33	135	0.401	13.2	241	34.5
1	0	3	1.6	2.8	1	0.57	120	0.377	12.8	87	40.9
1	1	6	6.9	1.7	0	4.06	84	0.276	17.5	538	33.5
1	0	9	2.8	1.6	0	1.75	101	0.308	13.2	269	37.9
1	1	6	3	1.5	0	2	145	0.431	12.7	176	38.1
1	1	8	3.3	0.8	0	4.13	72	0.223	12.1	306	33.1
1	1	7	4.7	1.9	0	2.47	133	0.415	13.2	265	40.8
1	0	6	3.7	1.7	0	2.18	71	0.218	14.8	308	33
1	1	6	5.91	1.3	0	4.55	134	0.384	12.3	184	42.7
1	1	6	5.4	2.1	1	2.57	141	0.427	13.5	349	32.9
1	1	8	3.5	1.2	0	2.92	110	0.342	13.5	152	35.3
1	1	6	2.4	1	0	2.4	130	0.385	12.9	179	41.7
1	1	5	3.9	2	1	1.95	95	0.28	13.4	246	35.9
1	1	8	12.6	1.3	0	9.69	101	0.299	16.5	260	16.6
1	1	7	3.1	1.9	0	1.63	148	0.444	13.1	203	43.4
1	1	6	6	1.3	0	4.62	107	0.325	11.8	197	33.2
1	1	7	2.1	2.1	1	1	143	0.404	11.7	76	35.2
1	1	9	3.9	0.2	0	19.5	80	0.238	14.4	202	19.3
1	1	8	3	1.8	0	1.67	133	0.409	13	134	35.8
1	1	7	3.6	1.4	0	2.57	144	0.433	13.1	219	43.3
1	0	6	2.8	1.4	0	2	116	0.361	13.2	230	36.9
1	0	5	4.8	0.9	0	5.33	60	0.213	17.9	281	35.4
1	1	8	5.2	0.9	0	5.78	118	0.361	12.4	187	34
1	0	5	1.8	2.3	1	0.78	87	0.295	50.5	326	44.3
1	1	7	5.9	1.2	0	4.92	115	0.36	14.3	227	37.3
1	0	6	4.8	0.7	0	6.86	132	0.402	13.1	69	44.3
1	0	5	3.7	1.7	0	2.18	121	0.379	14.3	152	38.8
1	1	7	5.2	1.7	0	3.06	139	0.401	12.4	283	41.1
1	1	7	2.9	1.5	0	1.93	146	0.425	12.8	163	36.7
1	1	6	2.6	0.9	0	2.89	130	0.367	13.6	343	43.9
1	1	8	2.9	1.3	0	2.23	82	0.252	18.4	129	29
1	0	7	4.8	2.2	1	2.18	120	0.357	12.2	205	42.1
1	1	8	5.7	0.6	0	9.5	80	0.264	18.8	110	36.8
1	1	6	6.1	1.3	0	4.69	100	0.326	15.5	360	36.9
1	0	4	2.2	1.2	0	1.83	136	0.405	12.6	215	43.6
1	0	5	2.9	1.1	0	2.64	85	0.262	13	177	30.3
1	1	5	3.5	1	0	3.5	130	0.393	12.3	169	44.8
1	1	7	3.8	2	1	1.9	155	0.463	12.5	167	39.9
1	1	9	2.9	1.8	0	1.61	122	0.356	13.2	157	33.5
1	0	6	3.3	2.7	1	1.22	111	0.348	13.4	242	35.3
1	1	7	2.6	1.7	0	1.53	127	0.391	13.3	400	33.8
1	0	7	4	1.5	0	2.67	112	0.338	12.5	210	32.8
1	1	7	5.9	1.3	0	4.54	175	0.517	13	203	44.1
1	1	7	3	1.2	0	2.5	106	0.315	12.7	174	38.1
1	0	6	1.9	1.7	0	1.12	135	0.415	12.8	218	46.8
1	1	7	2.1	1.2	0	1.75	145	0.421	13	216	43.4
1	0	5	5	1.1	0	4.55	129	0.384	14	224	42.1
1	0	7	1.8	1.5	0	1.2	126	0.389	13.1	147	41.9
1	1	6	3.4	1.1	0	3.09	162	0.463	12.7	187	48.2
1	0	7	3.5	1.9	0	1.84	148	0.44	12.2	234	45.3
1	0	6	6.2	1	0	6.2	98	0.319	16.2	292	29.3

1	1	7	1.6	0.8	0	2	52	0.175	20	55	21.8
1	1	7	3.2	1.6	0	2	158	0.454	13	183	35.4
1	1	7	5.1	1.1	0	4.64	157	0.451	12.1	266	47.2
1	0	7	2.8	1.5	0	1.87	153	0.453	12.5	254	44.4
1	0	5	2.5	0.8	0	3.13	110	0.342	14.9	131	25.9
1	1	7	5.6	1.1	0	5.09	54	0.158	14.8	190	29.6
1	0	6	1.5	1.7	0	0.88	84	0.265	21.2	226	37.1
1	1	6	4.4	2.6	1	1.69	155	0.454	12.2	259	43.5
1	0	7	4.1	1.8	0	2.28	120	0.374	13.5	298	43.6
1	0	7	9.4	0.7	0	13.43	118	0.361	15.2	168	37.9
1	0	6	4.6	1.5	0	3.07	127	0.373	12.2	250	39.2
1	0	8	1.9	1.1	0	1.73	80	0.256	14.8	172	32.4
1	1	5	3.1	3	1	1.03	154	0.45	12.8	158	38.2
1	1	6	8.1	2.1	1	3.86	122	0.377	13	380	41.1
1	0	7	2.5	1.7	0	1.47	134	0.395	12	285	42.8
1	1	8	2	3.2	1	0.63	144	0.428	13.4	291	36.7
1	1	5	3.5	1.2	0	2.92	148	0.444	11.9	210	42.6
1	1	7	6.9	2.1	1	3.29	102	0.309	11.9	162	43.7
1	0	6	4.75	2	1	2.33	122.5	0.3625	11.75	180.5	44
1	1	7	2.6	1.9	0	1.37	143	0.416	11.6	199	46
1	1	5	2.9	2.4	1	1.21	153	0.457	11.9	131	41.4
1	0	4	3.5	2	1	1.75	137	0.385	12.6	238	41.3
1	0	6	2	3.5	1	0.57	155	0.445	12.4	277	44.1
1	1	6	3.2	0.8	0	4	144	0.43	12	142	44.3
1	1	7	4.3	1.1	0	3.91	132	0.402	12.3	115	34.4
1	1	6	2.8	1.7	0	1.65	122	0.377	12.4	125	36.3
1	1	7	3.7	0.3	0	12.33	39	0.119	15.7	130	22.4
1	0	7	4.9	1	0	4.9	134	0.4	12.1	156	47
1	0	6	2.3	1.4	0	1.64	120	0.365	11.8	134	44.2
1	0	7	4.8	2.3	1	2.09	87	0.281	14.5	344	37.7
1	0	7	1.8	1	0	1.8	122	0.367	12.9	174	41.3
1	1	5	2.8	1.3	0	2.15	139	0.423	13.2	244	36.7
1	0	9	4.1	0.8	0	5.13	51	0.185	18.4	407	29.5
1	1	7	3.7	1.6	0	2.31	138	0.412	11.7	247	44.3
1	1	5	3.9	1.9	0	2.05	136	0.402	13.5	223	38.5
1	1	7	10.5	0.5	0	21	125	0.355	10.4	212	32.3
1	0	6	3.2	2.1	1	1.52	145	0.433	12.4	177	40.2
1	1	7	5.4	1.3	0	4.15	138	0.428	12.4	213	41
1	1	6	3.4	2	1	1.7	135	0.416	12.3	239	37.9
1	1	8	2.8	1.3	0	2.15	74	0.229	13.2	163	29
1	1	5	3.7	1.4	0	2.64	147	0.42	11.9	216	36.2
1	0	7	8.4	3	1	2.8	82	0.291	20	782	32.2
1	1	6	7.1	1	0	7.1	60	0.184	14.2	262	29.1
1	1	9	2.1	1.3	0	1.62	125	0.382	15.1	204	33.6
1	0	8	2.4	1.5	0	1.6	113	0.334	12.8	193	34.3
1	0	8	3.9	1	0	3.9	80	0.24	13.2	267	21.8
1	1	5	3.9	1.4	0	2.79	166	0.485	12.5	240	40.7
1	1	8	4.7	1.2	0	3.92	159	0.479	13	113	46.5
1	0	7	2.9	1.5	0	1.93	94	0.304	12.7	247	41.4
1	0	8	47.4	1.7	0	27.88	82	0.246	14.9	243	27
1	0	5	6.3	1	0	6.3	107	0.359	16.6	339	32.6
1	0	6	4.4	1.7	0	2.59	138	0.432	15.4	308	41.9
1	0	7	2.8	1.3	0	2.15	118	0.367	14.4	214	41
1	1	7	5.6	2	1	2.8	132	0.385	11.9	293	36.1

1	0	6	5.5	2.6	1	2.12	133	0.401	12.1	384	42
1	0	5	4.5	1.6	0	2.81	133	0.393	12.2	276	44.5
1	1	6	2.8	1.8	0	1.56	143	0.418	12.9	220	38.5
1	1	8	7.9	0.9	0	8.78	81	0.244	13.2	148	28.1
1	0	7	2.3	1.5	0	1.53	158	0.469	12.1	292	45.7
1	0	8	5.6	1.5	0	3.73	59	0.196	13.7	349	33.9
1	1	5	6	1.8	0	3.33	165	0.49	12.8	309	44.5
1	1	6	2.5	1.2	0	2.08	152	0.456	12.9	173	38.4
1	0	6	4.9	1.7	0	2.88	135	0.399	11.8	257	44.1
1	1	8	6.8	0.9	0	7.56	127	0.391	14.2	225	41
1	1	7	4.9	1.9	0	2.58	125	0.379	12.5	345	39.2
1	1	8	4.6	0.6	0	7.67	60	0.191	16.6	130	28.8
1	1	6	3.2	1.6	0	2	140	0.408	12.6	239	39.2
1	0	7	3.3	2.4	1	1.38	120	0.363	11.9	248	38.3
1	1	7	3.3	1.3	0	2.54	127	0.363	11.2	249	41.7
1	1	7	5	1.6	0	3.13	136	0.404	12.4	165	44.7
1	0	5	5.4	0.9	0	6	51	0.164	16.2	224	36.9
1	1	7	3.2	1.2	0	2.67	117	0.344	12.4	108	35.5
1	0	6	2.7	2.1	1	1.29	117	0.362	13.1	251	37
1	1	7	3.8	0.8	0	4.75	79	0.238	12	233	30.7
1	0	8	4.9	2.4	1	2.04	124	0.355	12.7	186	36.4
1	1	7	3.3	0.9	0	3.67	153	0.446	12.5	176	35.1
1	0	6	6.6	2.4	1	2.75	145	0.418	11.9	282	48.3
1	1	7	0.6	0.9	0	0.67	65	0.222	20.8	383	30.7
1	1	7	6.5	1.5	0	4.33	121	0.357	11.7	319	34.5
1	1	8	4.6	1.2	0	3.83	116	0.355	13	226	30.4
1	1	8	1.8	1.1	0	1.64	130	0.394	12.6	128	38
1	1	7	3.5	0.5	0	7	130	0.391	13.6	310	39.7
1	1	6	6.8	1.8	0	3.78	146	0.432	12.6	180	42.4
1	1	5	2.4	1.6	0	1.5	139	0.432	12.8	196	37
1	1	6	4.4	2.4	1	1.83	151	0.447	13.1	247	42.1
1	1	5	3.6	1.5	0	2.4	159	0.453	12.1	164	40.4
1	1	7	6.6	1	0	6.6	91	0.29	14.6	278	28.8
1	1	7	1.8	1.2	0	1.5	154	0.428	11.2	143	33.8
1	0	7	5.5	1.1	0	5	127	0.375	13.2	154	41.2
1	0	7	3.7	1	0	3.7	93	0.288	15.8	344	35
1	1	8	3.7	1.3	0	2.85	129	0.388	12.5	208	36.2
1	0	6	3.4	1.1	0	3.09	107	0.338	13.5	219	40.9
1	0	7	2.1	2.4	1	0.88	148	0.414	12.3	229	43.6
1	1	6	5.4	1.5	0	3.6	163	0.467	11.9	253	43.8
1	1	6	3.5	3.1	1	1.13	136	0.378	11.5	249	40
1	1	7	3.6	1.8	0	2	148	0.436	12.5	199	46.4
1	1	7	2.3	2.8	1	0.82	115	0.36	13.4	273	38.6
1	1	9	4.2	2	1	2.1	179	0.517	12.3	157	40.3
1	1	8	3.9	1.3	0	3	99	0.327	16	327	35.5
1	1	6	3.7	2	1	1.85	150	0.46	12.2	205	23.1
1	0	5	2.3	1.9	0	1.21	139	0.412	11.8	119	40.4
1	1	7	2.2	1	0	2.2	118	0.359	13.4	206	36.8
1	1	7	1.7	1.3	0	1.31	129	0.375	12.3	185	37
1	1	7	2.6	1.3	0	2	130	0.364	12.9	96	37.5
1	0	4	2.2	1.1	0	2	127	0.389	13.4	216	46
1	1	8	4.7	1.6	0	2.94	134	0.395	11.8	195	32.1
1	0	9	5.4	1.1	0	4.91	77	0.232	13.9	207	21.3
1	1	6	1.6	0.8	0	2	73	0.225	14.9	238	30.9

1	0	5	2.6	1.55	0	1.785	74	0.228	15.2	278	45.6
1	1	7	3.6	2.3	1	1.57	75	0.231	15.5	318	37.5
1	1	8	1.9	0.8	0	2.38	122	0.358	12.4	251	34.6
1	1	6	4	1.1	0	3.64	74	0.246	15.5	283	28.1
1	0	5	4.4	1.8	0	2.44	121	0.385	12.5	328	41.6
1	0	8	1.4	1	0	1.4	113	0.329	12.2	58	37.4
1	0	5	4.3	1.3	0	3.31	99	0.302	13.3	160	36.1
1	1	6	4.3	0.9	0	4.78	136	0.401	12.8	197	42.1
1	1	7	4.8	0.4	0	12	119	0.37	15.1	199	37.8
1	1	5	2.6	1.3	0	2	136	0.402	13.2	273	43.5
1	0	8	5	2.3	1	2.17	131	0.389	12.8	228	35.3
1	0	6	3.8	1.5	0	2.53	129	0.387	12.8	241	41.7
1	0	6	3.7	3.1	1	1.19	154	0.439	11.8	276	42.4
1	1	6	6.8	1.3	0	5.23	113	0.329	19.8	489	35.5
1	0	8	2.4	1	0	2.4	130	0.372	12.6	170	41.6
1	1	7	3.6	1.7	0	2.12	138	0.419	13.2	110	42.6
1	0	6	2.9	1.2	0	2.42	128	0.385	11.7	161	41.5
1	1	6	2.3	1.5	0	1.53	145	0.423	10.9	181	37.4
1	1	8	3	1.1	0	2.73	143	0.428	12.9	178	44.1
1	1	6	3	2.3	1	1.3	147	0.427	12.2	266	12.3
1	0	7	5	1.7	0	2.94	123	0.367	12.1	288	37.8
1	1	7	4.9	1.2	0	4.08	131	0.386	13	146	35.8
1	1	8	5.2	2.4	1	2.17	143	0.41	14.5	210	36.9
1	1	8	2	1.1	0	1.82	89	0.273	15.1	156	34.8
1	1	7	2.9	1.7	0	1.71	125	0.374	13.7	337	38.3
1	1	9	3.1	1	0	3.1	106	0.322	16.4	93	33.6
1	1	7	2.9	1.6	0	1.81	123	0.326	12.3	247	41.1
1	1	7	3.1	1.2	0	2.58	122	0.353	12.1	200	38.2
1	1	7	6	2.3	1	2.61	104	0.322	15.1	520	36.5
1	1	6	2.8	1	0	2.8	134	0.389	12	164	32.3
1	0	5	7	2.1	1	3.33	133	0.382	11.8	352	32.1
1	0	6	2.6	1.3	0	2	114	0.341	13.8	76	43.4
1	0	5	2.3	1.5	0	1.53	77	0.231	12.4	221	30.9
1	0	7	3.7	1.6	0	2.31	143	0.433	12.2	313	44
1	1	6	3.1	2	1	1.55	144	0.431	12.1	124	39.3
1	1	7	4.6	1.1	0	4.18	125	0.381	14.2	383	28.5
1	1	8	5.1	1.4	0	3.64	76	0.267	17.1	478	22.4
1	1	8	2.2	1.6	0	1.38	94	0.316	17.2	334	30.6
1	0	9	4.7	1.1	0	4.27	118	0.351	11.9	408	32.7
1	1	7	5.3	2.1	1	2.52	89	0.263	12.7	378	36.9
1	1	7	4.2	2.3	1	1.83	140	0.42	12.6	244	41.9
1	1	7	5.2	1.1	0	4.73	119	0.371	12.8	227	28.8
1	0	7	1.9	1.5	0	1.27	128	0.382	12	227	37.7
1	1	5	2.6	1.6	0	1.63	141	0.433	12.9	133	29.2
1	1	5	3.1	2	1	1.55	87	0.257	12.9	132	32.8
1	0	7	2	1.2	0	1.67	115	0.35	13.7	255	30.5
1	0	7	3.2	1.3	0	2.46	119	0.342	12.3	190	41.5
1	0	3	5.7	0.8	0	7.13	127	0.368	13.4	387	33.1
1	1	7	3	2.2	1	1.36	143	0.412	12.8	188	38.5
1	1	8	4	1.4	0	2.86	122	0.364	13.8	155	30.4
1	0	6	6	1.7	0	3.53	133	0.399	14	375	37.7
1	1	7	2.9	1.6	0	1.81	146	0.431	12.5	176	38.9
1	1	5	3.8	0.9	0	4.22	127	0.367	12.1	172	39.9
1	1	7	5.8	1.5	0	3.87	136	0.415	13	166	39.6

1	1	7	3.6	1.1	0	3.27	116	0.359	13.9	177	41.8
1	1	7	3.9	2.1	1	1.86	150	0.438	13.4	149	37.3
1	1	7	2.5	1.7	0	1.47	169	0.501	12.6	173	47
1	1	7	4.1	1	0	4.1	112	0.34	13.4	175	39
1	1	7	3.2	1.2	0	2.67	125	0.371	12.6	237	33.9
1	1	7	3.3	2	1	1.65	75	0.267	17.5	202	37.5
1	1	7	3.2	1.5	0	2.13	139	0.407	11.6	193	44.6
1	1	7	5	1.3	0	3.85	151	0.437	13.1	255	42.1
1	1	9	9.5	1.1	0	8.64	92	0.275	14.6	265	28.2
1	1	8	6.5	1.4	0	4.64	119	0.362	12.9	239	22.6
1	1	7	3.1	0.5	0	6.2	104	0.318	14.7	110	35.8
1	1	7	6.2	1.2	0	5.17	95	0.29	11.8	140	32.1
1	1	7	3.9	1.6	0	2.44	123	0.365	12.3	227	40.6
1	1	7	5	2.4	1	2.08	139	0.407	12.6	337	41.6
1	0	7	2.5	1.8	0	1.39	110	0.347	15.3	218	45.6
1	1	6	6	1.1	0	5.45	118	0.331	15.7	327	36
1	1	6	3.3	1.4	0	2.36	153	0.448	12.5	218	38.7
1	0	8	2.5	1	0	2.5	96	0.299	18.7	192	30
1	1	7	4.3	0.9	0	4.78	140	0.399	12.3	248	36.8
1	1	6	3.6	2	1	1.8	153	0.447	13	269	41.8
1	1	6	2.7	2.7	1	1	160	0.468	11.6	158	41
1	0	6	4.2	1.6	0	2.63	124	0.366	12.1	247	38.7
1	0	6	3	1.3	0	2.31	132	0.4	13.5	178	39.8
1	1	6	1.9	1.1	0	1.73	144	0.409	12.5	215	36.8
1	1	5	2.9	2.2	1	1.32	146	0.429	12.5	125	37.4
1	1	6	3.5	1.1	0	3.18	85	0.263	13.1	320	26.3
1	1	6	1.5	1.8	0	0.83	131	0.386	13.7	242	37.3
1	1	7	4.5	1.2	0	3.75	103	0.323	15.3	187	39.3
1	1	7	2.8	2	1	1.4	141	0.419	11.8	214	39.1
1	0	5	7.6	1.2	0	6.33	103	0.318	14	455	28.4
1	1	7	2.8	2.1	1	1.33	96	0.301	15.3	323	37.4
1	1	7	1.6	1	0	1.6	88	0.265	13.1	251	36.1
1	1	7	4.5	1.2	0	3.75	144	0.429	13.2	166	42.7
1	1	5	9.9	1.1	0	9	91	0.282	16.1	553	30
1	1	6	3.5	2.2	1	1.59	154	0.437	12.5	193	37.3
1	0	9	4.6	0.9	0	5.11	88	0.274	13.9	221	22.9
1	1	6	3.1	1.5	0	2.07	60	0.21	15.6	313	36.5
1	1	8	5	1.4	0	3.57	108	0.315	11.1	267	36.5
1	1	7	8.8	1.2	0	7.33	93	0.304	16.1	246	31.6
1	1	8	4	2.9	1	1.38	145	0.418	12.3	262	42.9
1	1	7	3.9	2.1	1	1.86	122	0.369	12.7	194	34.1
1	1	8	3.3	1.3	0	2.54	87	0.292	15.7	283	39.2
1	0	6	4.2	1.2	0	3.5	140	0.417	12.2	222	42.9
1	1	7	2.5	1.1	0	2.27	137	0.374	11.5	165	35.9
1	1	7	2.6	1.5	0	1.73	118	0.347	14.1	145	33.3
1	1	6	3.2	2.3	1	1.39	135	0.388	13.6	186	38.3
1	1	5	6.6	1.1	0	6	138	0.416	13.2	253	30.3
1	0	5	4.9	2.3	1	2.13	156	0.451	14.7	265	46
1	1	7	3.9	1.6	0	2.44	139	0.421	15.9	243	41.2
1	1	8	2.2	2.2	1	1	125	0.366	13.2	198	40.2
1	1	4	2.4	1.9	0	1.26	150	0.44	11.6	286	43.8
1	0	8	4.4	2.4	1	1.83	149	0.437	12.5	284	41.8
1	1	7	4.3	0.7	0	6.14	157	0.461	13.1	186	41.7
1	0	7	4.5	0.9	0	5	132	0.398	13.2	217	31.9

1	1	5	4.6	2	1	2.3	143	0.433	13	297	37.6
1	1	5	3.6	1.1	0	3.27	145	0.435	12.2	268	44.2
1	0	7	2.5	2.3	1	1.09	99	0.307	15.9	277	40.6
1	1	6	3.6	2	1	1.8	156	0.447	12.8	171	43.4
1	1	6	3.9	2.1	1	1.86	117	0.361	14.3	293	39.7
1	0	6	3	1.5	0	2	132	0.392	118	215	45.8
1	0	8	2.3	2	1	1.15	124	0.365	12.6	191	34.3
1	0	8	6.4	1.4	0	4.57	63	0.204	16.5	330	31.1
1	1	6	6.1	2.7	1	2.26	146	0.432	13.1	228	40.5
1	1	6	4.6	1.9	0	2.42	112	0.354	16.8	275	39
1	1	8	3.5	1.2	0	2.92	139	0.398	12.8	119	44.3
1	1	6	3.1	2.2	1	1.41	149	0.448	13.9	210	43.2
1	0	7	4.5	2.3	1	1.96	131	0.405	14.3	318	45.4
1	1	7	4.4	2.1	1	2.1	154	0.448	11.4	241	41.7
1	0	8	4.3	1	0	4.3	96	0.309	13.9	241	30.7
1	0	6	3.5	1.5	0	2.33	130	0.4	13.9	205	43
1	0	7	0.9	1.5	0	0.6	128	0.391	13.4	100	39
1	1	7	3.1	1.4	0	2.21	128	0.405	13.8	220	42.9
1	1	7	7.8	1.1	0	7.09	69	0.226	21.4	271	26.1
1	1	7	10	0.7	0	14.29	138	0.43	13.2	230	35.2
1	1	6	4.5	1.4	0	3.21	145	0.436	12.3	167	42
1	1	6	3.8	2.6	1	1.46	165	0.475	12.3	219	39
1	0	5	2.6	1.6	0	1.63	150	0.45	12.2	193	44.5
1	0	8	2.5	0.4	0	6.25	118	0.368	12.5	212	32.4
1	0	6	2	2.2	1	0.91	134	0.407	12.7	280	43
1	1	6	2.6	1.4	0	1.86	115	0.329	12	298	29.3
1	1	6	5.5	0.7	0	7.86	139	0.412	14.5	185	33.6
1	1	7	3.3	1.4	0	2.36	127	0.38	13.6	136	36.6
1	0	6	2.6	1.2	0	2.17	117	0.351	13.2	190	44.4
1	1	7	7.2	1.7	0	4.24	125	0.375	12.5	204	40.4
1	0	5	3.8	1.1	0	3.45	126	0.385	12	218	39.7
1	1	7	4.5	1.5	0	3	118	0.363	13.1	312	40.4
1	1	6	3.8	2	1	1.9	157	0.453	11.8	316	43.6
1	1	7	2.7	1.1	0	2.45	57	0.204	18.7	257	40
1	1	6	3.2	1.9	0	1.68	143	0.422	12.8	161	39.9
1	1	6	7.4	0.6	0	12.33	68	0.232	22	142	30.8
1	1	3	2.1	0.9	0	2.33	94	0.28	15.5	182	40.1
1	1	7	3.5	0.8	0	4.38	127	0.367	13.5	180	38.4
1	1	7	2.5	1	0	2.5	80	0.271	16.6	308	35.9
1	1	6	3.1	2.1	1	1.48	164	0.468	12	263	39.7
1	1	6	3.4	1.3	0	2.62	118	0.368	12.8	197	33.3
1	1	7	2.4	1.7	0	1.41	132	0.391	12.2	183	38.2
1	0	5	2.1	2	1	1.05	119	0.374	12.8	217	42.6
1	0	7	3.9	1.2	0	3.25	99	0.321	16	262	35.2
1	1	5	3.7	2.1	1	1.76	141	0.412	12.3	197	37.6
1	1	7	2.7	2.9	1	0.93	155	0.449	12.1	232	44.7
1	0	6	3.9	1.6	0	2.44	142	0.411	11.6	250	44.7
1	0	7	3	2	1	1.5	112	0.359	12.8	213	38.9
1	0	6	1.9	1.5	0	1.27	137	0.4	12.1	144	43.8
1	0	6	5.5	1.7	0	3.24	112	0.344	14.7	135	45.6
1	0	8	7.1	1.3	0	5.46	104	0.297	12.5	166	34.5
1	0	7	3.5	4	1	0.88	115	0.366	12.7	313	42.4
1	0	7	2.8	1.8	0	1.56	130	0.4	14.5	256	397
1	1	6	3.3	1.1	0	3	147	0.421	12.5	139	41

1	0	6	3.5	0.9	0	3.89	77	0.26	18.9	172	40.2
1	1	6	2.4	1.3	0	1.85	148	0.426	15.7	158	42.4
1	0	6	9.6	2.2	1	4.36	131	0.393	13.1	304	40.4
1	1	7	7.2	1.6	0	4.5	133	0.389	11.7	231	42.8
1	0	7	2.8	2.5	1	1.12	121	0.382	14.2	242	40.3
1	1	5	3.6	3.3	1	1.09	140	0.408	12.8	166	43.3
1	1	5	3.7	1.1	0	3.36	99	0.318	14.4	393	35.3
1	1	6	4.1	1.7	0	2.41	136	0.408	11.9	208	34.9

alt	tb	cr	tg	chol	hdl	ldl	lpa	cea	ca125	ca199	ca724
17	9.5	70.7	1.11	6.28	1.6	3.56	314	1.5	16	12.7	1.3
24	22.5	101.9	1.23	3.82	1.08	2.27	236	3.2	15.7	11.1	2.05
90	13.6	68.6	1.69	3.39	0.83	2	87	4.1	15.4	30.9	2.8
23	16.5	61.3	2.92	6.51	1.09	3.76	134	1.2	15.1	7.1	3.55
22	6.2	82.3	1.62	6.1	1.3	3.89	127	2.1	14.8	13.8	4.3
28	9.15	71.4	2.09	5.255	1.205	2.95	175	1.86	12.5	10.8	9.23
34	12.1	60.5	2.56	4.41	1.11	2.01	223	1.63	10.2	7.93	14.1
11	17	77.3	1.07	3.62	1.14	2.1	88	1.4	7.9	5	19.1
10	15.1	66.5	1.05	5.18	1.47	2.95	719	1.3	16.8	0.9	18.03
54	15.5	78.8	4.1	4.07	0.8	1.68	343	0.4	25.7	6.4	16
21	8.9	63.8	2.48	4.46	1.17	2.45	150	11	34.6	18.2	15.9
23	15.3	73.1	1.87	5.39	1.03	3.6	36	7	9.8	46.6	1.5
17	11.7	66.4	1.75	5.17	1.28	3.19	37.3	5.06	11.16	34.6	1.32
17	22.9	59.7	1.63	4.96	1.53	2.79	38.6	3.13	12.52	22.6	1.14
17	34.1	83.6	1.51	4.75	1.78	2.39	40	1.2	13.88	10.6	0.96
12	9.9	77.2	0.76	5.96	1.58	3.7	423	0.9	15.24	8	0.78
11	15.7	82.6	1.98	4.12	1.26	2.18	93	3	16.6	18.8	0.6
16	14.3	72.4	1.4	5.4	1.42	3.31	112	1.1	13.15	3.7	1
20	16.6	84.6	2.88	4.2	0.95	1.9	23	2.8	9.7	6.2	1.4
53	19.6	74.8	2.35	4.93	1.11	2.85	173	1.1	13.9	13.8	9.8
102	12.1	96	3.82	6.75	1.5	3.89	57	1.2	11.05	6	8.8
13	8.8	68.3	1.93	4.77	1.05	2.61	73	1	8.2	1	7.8
29	16.7	95.7	1.14	3.43	1.29	1.59	138	1.5	4.6	16.1	1
12	9.2	58.4	1.49	3.6	1.12	1.95	36	3.5	9	40.1	4
14	10.8	63.1	1.205	3.895	1.505	1.96	273.5	2.55	12.2	24.25	6.05
16	12.4	67.8	0.92	4.19	1.89	1.97	511	1.6	15.4	8.4	8.1
41	6.7	66	2.19	4.05	0.99	2.41	40	1.7	13.775	8.4	6.8
5	9	66.2	1.78	4.1	1.15	2.32	397	1.8	12.15	8.4	5.5
13	22.6	58.2	1.37	6.29	1.56	3.76	272	1.9	10.525	48.2	4.2
24	32.6	71.3	1.27	3.86	1.37	2.1	34	2.6	8.9	8.5	2.9
12	20.2	66.9	0.62	4.49	1.75	2.35	43	3.05	10.3	10.5	1.95
38	14.8	77.4	1.1	4.55	1.22	2.9	17	3.5	11.7	12.5	1
11	12.2	63.6	1.11	3.97	1.21	2.25	82	1.7	8.1	8.1	0.8
7	12.1	57.8	1.1	5.14	1.48	3.18	270	1	5.1	4.9	4.4
22	9.5	58.9	1.1	5.43	1.6	3.19	118	0.4	49	31.3	4
7	10.1	87.3	2.51	4.09	0.84	2.17	29	2.1	8.8	5.1	1
26	9.8	76	1.64	4.5	0.99	2.75	55	1.7	12.2	21.2	1
21	20	81.3	2.11	5.38	1.22	3.34	252	3.1	12.1	1.7	4.4
21	14.75	74.7	1.725	5.535	1.31	3.345	490.5	1.8	10.05	13	4.25
21	9.5	68.1	1.34	5.69	1.4	3.35	729	0.5	8	24.3	4.1
16	14.7	92.3	1.38	4.19	1.22	2.33	89	2.3	6.1	10.9	3.7
59	18	78.8	1.28	4.65	0.93	2.88	83	0.9	21	9	6.4
13	9.6	53.7	1.32	3.92	0.94	2.41	172	2.6	17	45.5	4.55
78	18	79.3	0.85	5.06	1.43	2.96	34	4.3	13	82	2.7
32	8.7	71.2	1.66	4.56	1.22	2.49	127	0.6	9.2	6.8	1.3
24.5	10.25	67.5	1.435	4.36	1.21	2.445	297.5	0.65	9.65	8.05	1.35
17	11.8	63.8	1.21	4.16	1.2	2.4	468	0.7	10.1	9.3	1.4
60	17.6	90.4	2.97	7.32	0.95	6.37	251	0.5	11.35	8.95	1.05
10	9.4	66	0.85	4.29	1.43	2.43	34	0.3	12.6	8.6	0.7
11	24.15	69.15	0.94	4.59	1.8	2.265	94.5	0.7	8.8	15.9	0.85
12	38.9	72.3	1.03	4.89	2.17	2.1	155	1.1	5	23.2	1
17	15.2	81.6	1.2	5	1.09	3.3	136	1.9	12.5	6.9	10.3
23	17.5	63	0.59	4.76	1.63	2.76	73	1	24.6	14.3	1.6

24	14	65.1	1.07	5.38	1.62	2.98	96	1.8	15	9.3	0.4
18	8.8	114.7	1.05	5.06	1.39	3.67	452	1.4	16.8	8.85	0.65
39	16.2	65	1.91	6.75	1.21	4.37	808	1	18.6	8.4	0.9
14	12.4	119.7	0.61	4.36	2.12	1.76	100	3.5	7.3	47.7	1.6
25	9.3	76.7	1.58	5.03	1.24	2.85	317	1.6	11.2	1	28.3
24	14.3	74	9.67	5.685	0.925	2.05	183.5	2	11.55	13	14.85
23	19.3	71.3	17.76	6.34	0.61	1.25	50	2.4	11.9	25	1.4
21	10.8	66.8	8.95	6.11	0.84	1.47	35	1.8	16.9	7.3	0.9
22	16.1	90.1	1.01	4.18	1.01	3.17	354	2.5	13.6	12.5	0.8
10	13	65	0.78	6.09	1.55	3.76	673	1.3	10.3	5.4	0.7
18	9.1	49.4	1.02	5.46	1.42	3.61	447	1.6	82	251.1	5.9
19	26.4	99.7	2.6	6.01	1.285	3.165	613	1.2	45.65	5.4	3.5
8	10.6	65.9	4.18	6.56	1.15	2.72	779	1.9	9.3	12.2	1.1
24	14.1	60.6	1.93	4.85	1.25	2.87	338	1.5	11.1	17.4	0.7
17	8.6	72.5	1.05	4.26	1.51	2.17	72	2.6	5.1	13.6	0.725
42	23.3	92.5	1.13	4.98	1.24	3.5	95	2.23	5.86	12.13	0.75
29	18.1	70.9	1.95	5.14	1.18	3.18	80	1.86	6.6	10.66	0.775
19	13	110	0.69	4.77	1.33	2.89	489	1.5	7.4	9.2	0.8
70	21.5	75.8	3.82	4.9	1.01	2.4	44	1.1	13.4	8.2	3.6
26	26.8	56.4	10.88	6.39	0.86	1.73	849	3.2	9	1.5	39.6
29	8.8	75.3	1.17	4.26	1.3	2.5	33	1	15.9	10.8	20.3
20	23.2	72.5	0.91	5.76	1.62	3.68	259	3.6	22.8	25.7	1
33	7.9	61.3	1.68	3.15	1.1	1.58	62	3.2	20.5	5.5	24.5
23	11.8	79.9	1.5	4.07	0.84	2.72	249	2	11.4	20.1	5.7
14	11.9	72.7	1.84	8.02	1.48	4.9	203	1.6	12.36	15	4
12	9.5	56.6	0.77	5.08	1.55	2.78	653	1.3	13.3	9.96	2.43
15	15.9	64.1	0.92	4.74	1.45	2.72	86	1	14.3	4.9	0.8
15	14.5	66	0.78	4.25	1.51	2.03	11	1.6	6.2	12.1	8.2
13	13.7	93.8	1.13	6.02	1.3	4.1	216	0.9	6.5	5.9	1.3
20	11.9	78.5	0.96	3.92	1.16	2.29	1218	2.3	8.9	10.5	4.1
33	11.3	89.8	3.57	6.81	1	4.2	55	1.4	20.8	6.7	0.4
38	8.5	56.7	1.53	5.69	1.32	3.73	256	2	15.1	28.4	2.2
14	14.2	83	1.84	6.77	1.14	4.35	298	2.5	13.3	24.6	1.1
8	8	54.8	1.21	4.96	1.63	2.78	42	2.9	23.1	11.3	3.3
10	10.6	62.9	1.19	4.24	1.74	2.07	26	1.2	19.1	18.3	1.9
10.5	14.5	69.1	1.065	4.055	1.385	2.245	77.5	1	13.65	10.95	1.45
11	18.4	75.3	0.94	3.87	1.03	2.42	129	0.8	8.2	3.6	1
10	6.3	67.4	1.86	4.77	0.97	2.72	140	0.9	15.8	5.4	1.8
12	14	66.3	0.63	4.41	1.56	2.28	657	0.8	17.2	7	1.7
16	13.4	93.5	1.65	4.67	0.83	3.28	350	2.5	6.8	10.9	0.8
16	11.7	90.1	1.19	4.29	0.98	2.51	510	3.9	11.1	6.05	1.45
17	14.1	58.9	0.92	6.06	2.23	3.01	69	5.3	15.4	1.2	2.1
14	10.7	62.6	0.78	4.42	1.15	2.67	16	2.4	12.2	12.7	2
46	9.6	74.9	1.45	4.86	1.09	2.92	83	0.9	4.3	11.7	1.2
45	16.3	78.4	2.53	5.21	1.33	3.09	337	1.6	12.2	9.8	2.5
16	9.2	72.2	1.13	5.55	1.19	3.84	54	1.6	19.2	7.9	1.3
60	12.4	74.7	2.71	6.04	1.39	3.51	11	1.7	14.9	7.4	6.2
27	15.7	94.6	0.99	5.26	1.53	2.9	208	1.6	6	7.3	0.9
30	12.1	94.7	2.73	4.72	1.06	2.74	2	2.4	12	10.3	6.1
22	17.9	98.1	1.43	4.84	1.14	2.99	67	1.3	7.3	5	5.2
23	12.4	89.9	1.53	4.5	1.48	2.22	821	1.8	10.3	5.3	1.1
18	10	84.8	2.225	4.79	1.32	2.675	524.5	9.85	8.25	9.65	3.75
13	7.6	79.7	2.92	5.08	1.16	3.13	228	1.9	6.2	14	6.4
8	14.2	77.3	1.18	5.89	1.85	3.13	76	1	5.7	23.9	3.2

20	12.3	77.1	1.32	4.59	1.02	2.73	62	1.2	18.8	5.8	0.8
47	21.1	93.3	3.41	6.84	1.12	4.26	103	1.26	16.2	7.6	0.76
12	8.1	63.4	0.6	4.64	1.8	2.24	165	1.33	13	9.4	0.73
12	7.5	56.9	1.1	5.78	1.58	3.41	28	1.4	11.1	11.2	0.7
30	10.6	80.5	2.25	5.39	1.08	2.99	114	3.1	5.5	9.5	18.2
8	9.9	85.7	0.7	3.88	1.26	2.32	85	2	7.3	7.1	12.5
20	17.2	94.3	1.01	4.35	1.03	2.68	46	2	2.5	14.4	2.1
19	8.4	61.4	1.65	6.03	1.1	3.6	124	2	16.9	10.3	0.8
17	13.6	62.1	1.92	4.9	1.25	2.58	35	1.4	14.7	21.9	30.5
21	10.3	85.5	5.11	4.71	0.77	2.12	11	2.5	10.1	18.2	1.1
20	17.1	73.4	2.2	5.37	1.47	2.82	112	0.7	11.7	3.6	3.9
30	12.7	71.2	2.565	4.93	1.15	2.445	105	2.8	12.5	11.4	1
22	8.3	79.7	2.93	4.49	0.83	2.07	98	1.6	8.9	7.6	0.8
21	12.1	60.8	1.57	6.67	2.27	3.13	64	4.7	14.9	31.4	1.9
27	11.5	64.1	0.62	4.79	1.88	2.14	39	1.5	17.4	21.9	1.9
14	10	61.5	0.83	4	1.13	2.32	19	1.5	15.1	9	0.5
29	14.6	78	2.07	4.6	0.99	2.79	60	3	15.8	23.4	1.5
18	19.9	78.1	0.66	4.62	1.32	2.69	444	1.6	14.6	7.7	0.9
17	19.6	55	0.92	5.48	1.54	3.13	45	1.7	11.1	19.9	0.9
17	13.7	67.5	0.53	4.86	1.88	2.98	40.5	0.8	9.7	8	8.3
67	9	54.8	1.13	2.84	0.7	1.78	36	2	6.1	44.3	0.8
16	12.5	57.7	0.72	5.71	1.4	3.6	1065	0.5	11.9	8.3	0.7
33	12.8	80.2	0.74	4.48	1.56	2.18	210	2.5	10.5	20.3	2.5
16	9.7	59.8	1.05	5.67	1.45	3.23	612	1.7	14.3	15.7	2
15	12.1	67.5	0.69	3.53	1.21	1.95	86	0.9	18.1	11.1	1.5
21	21.7	77.8	6.72	1.32	2	3.66	41	2.45	13.95	6	95.65
31	18.1	99.9	0.96	4.66	1.31	3.01	406	4	9.8	0.9	189.8
9	19.1	61.6	0.57	4.92	2.13	1.88	114	1	5.2	9.8	23
26	10.1	66.8	1.94	5.64	1.03	3.73	121	2.75	10.65	20.25	12.7
19	10.7	84.2	1.55	4.97	1.21	2.96	324	4.5	16.1	30.7	2.4
17	14.3	63.3	1.37	5.96	1.42	3.39	40	3.6	23.1	32	2.9
46	24.8	98.3	1.99	4.24	0.87	2.35	394	1.6	12.8	9.3	6.7
18	10.2	63.1	0.58	4.58	1.74	2.03	53	2.3	13.9	21.3	0.9
16	14.5	83.9	1.32	3.96	1.21	2.18	5	9.5	5.3	47	5.6
11	11.9	57	1.02	4.75	1.24	3.03	94	5.75	9.75	27	23.25
21	17.4	77.7	0.57	3.92	1.13	2.28	40	2	14.2	7	40.9
18	30.3	67.4	2.74	3.81	0.77	1.91	28	3.9	13.1	1.1	44.9
28	9.9	61	1.63	4.99	1.06	3.11	71	2.6	14	22.5	3.2
17	15.5	59.9	1.17	4.78	1.36	2.87	18	1.4	9.5	10.8	4.8
17	5.9	70.1	1.06	4.29	1.39	2.28	112	1.2	13.8	10.3	8.5
12	9.6	61.8	0.95	3.74	1.26	1.86	285	1	18.1	9.8	12.2
15	14.4	50.3	0.67	3.78	1.41	2.18	40	4.1	6.5	18.9	1.1
28	7.1	70.1	0.57	3.55	1.58	1.46	106	5	9.4	7.4	2.3
19	7.4	71.1	0.62	4.86	1.6	2.61	55	1.3	6.3	6.2	5.2
16	8.1	75.3	1.06	3.62	1.32	1.83	123	1.6	5.8	1.9	4.7
25	10	65.2	1.66	4.23	1.17	2.42	448	2.2	7	1.7	50
15	14.9	75.1	1.06	4.95	1.21	3.51	68	2.7	4.8	8.8	1
25	10.7	62	0.59	4.4	1.7	2.58	126	2.5	10.9	1.5	6.9
30	17	78.9	1.19	3.87	1.18	1.98	157	2.4	20.1	0	2.7
40	12.5	77	1.16	3.97	0.79	2.62	49	1.9	6.3	9.8	2.1
11	10.3	57.6	2.12	6.86	1.15	4.88	104	1.4	10.7	13.2	1.3
22	14.8	65	0.92	5.91	1.63	3.25	283	1.1	14.7	9.2	53.2
27	20.1	68.3	2.51	7.5	1.34	4.57	103	2.3	4.7	7.8	5.9
23	9.9	64.4	0.64	5.03	1.4	2.77	67	1.1	6.3	4.4	19.1

26	26.3	78.3	0.95	4.49	1.04	2.62	474	1.3	5.9	7.6	1.1
27	7.7	81.8	0.76	4.63	0.89	3.05	14	2	8	8.3	11
12	16.9	66.4	1.12	4.76	1.23	2.43	184	2.8	7.5	19.7	2.8
15	7.4	64.8	0.89	4.17	1.71	1.95	300	0.7	23.2	16.2	3.3
13	11.3	62.9	1.36	5.95	1.43	3.49	206	1.5	14.1	6	2.3
10	19.1	77.6	1.44	4.37	1.06	2.42	67	1.5	5.9	14.4	2.3
47	14.9	84.5	1.9	5.64	1.19	3.59	22	3.4	15.9	16	0.8
13	6.7	63.1	2.28	6.12	1.05	3.78	69	1	5.3	4	1.2
11	9.4	65.7	0.95	4.02	1.07	2.5	200	1.8	3.9	8.6	0.9
69	10	76	1.73	4.84	1.01	2.76	13	1.5	8.8	4.9	1.8
22	7.2	83.1	1.48	4.83	0.96	3.28	68	1.6	11	8.9	1.1
12	14.6	63.4	0.84	5.06	1.55	2.93	78	0.6	17.1	33	7
16	12.5	79.7	2.27	5.54	1.3	3.04	197	2.2	9.3	3	1.7
16	7.9	68.4	0.56	5.83	3.43	2.05	521	1.9	13.6	8.1	2
16	11.2	75.1	0.91	5.22	1.37	3.05	276	1.4	20.1	4.3	1.7
6	10.1	55.8	3.73	2.78	0.73	0.83	34	1.2	7.9	8.7	0.9
24	16	56.7	0.97	5.7	1.38	3.79	477	0.7	5.3	12.1	2
20	12.4	56.4	0.48	5.29	2.17	2.63	105	1.9	7.1	12.3	9.5
13	11.8	82.3	1.4	7.08	1.62	4.27	45	1.1	12.6	11.1	1.2
8	9.1	61.5	0.66	3.59	1.51	1.86	12	0.3	10.1	7.6	1
36	20	72.6	1.11	5.53	1.19	3.81	91	3.2	18.3	0	4.5
17	17.5	74.6	1.22	4.73	1.26	3.04	77	1.2	13.4	6.9	15.5
12	9.8	56.4	1.15	4.36	1.42	2.57	112	1.3	5.5	10.1	6.1
12	20.5	76.9	1.3	5.32	1.52	2.96	41	3.9	10.4	22.2	4.2
26	7	84.7	2.77	4.68	0.77	2.69	75	2.5	3.8	0	17.9
10	6.5	75.1	0.92	6.32	3.01	2.84	329	0.8	89.4	24.2	5.6
16	6.7	66.7	0.89	5.09	1.3	3.08	165	0.2	8.3	6.7	3.3
14	15.8	76.3	1.07	6.58	1.6	4.4	182	2.7	10.4	7.1	1.8
17	8.8	63.6	1.11	4.88	1.46	2.85	57	1.4	8.25	5.2	8
12	13.9	75.9	0.56	3.54	1.25	2.22	256	1.1	6.1	9	14.2
14	11.3	54.3	0.69	4.77	1.68	2.52	12	0.8	16	5.9	35.1
34	17	91.7	2.06	4.09	0.97	2.19	40	1.7	9.5	9.9	2.1
13	16.2	81.3	1.38	5.08	1.13	3.07	215	1.3	9.1	11	16.6
14	14	61.6	1.26	5.79	1.24	4.22	448	1.9	8.9	9.8	3
20	13.4	87.9	1.86	5.11	1.17	3.38	87	1.3	13.6	2.8	5.5
9	20.2	68.6	1.44	4.65	1.17	2.89	181	3.3	15.9	21	1
30	10.3	78.1	1.83	6.32	1.36	3.77	371	1.3	9.8	7.2	51.2
11	5.7	61.2	1.29	4.72	0.98	2.94	99	0.7	11.7	2.2	3.6
6	16.9	60.5	0.45	4.24	1.61	2.14	228	5.2	11	21.5	2.5
25	9.1	69.3	0.97	3.99	1.03	2.57	10	0.9	10.3	1.9	1.4
8	12.9	71.9	1.42	5.1	1.2	3.14	305	1.5	20.9	7.3	2.5
18	13.2	63.8	0.51	3.67	1.75	1.58	67	6.9	16.05	20.2	2.2
23	10.4	47.9	2.48	5.38	1.26	3.25	29	12.3	11.2	33.1	1.9
17	15.1	97.5	1.51	6.15	1.16	4.08	127	2.4	14.6	17.6	3.3
11	7.1	56.9	0.67	4.03	1.52	2.13	91	1.2	10.8	16.6	3.1
17	9.6	60.3	2.36	6.47	1.38	4.34	382	1.1	10.9	7.2	1.8
18	20.1	78.4	1.73	5.95	1.27	3.73	208	3.1	14	17.5	0.9
36	20.3	63.8	1.02	4.08	1.04	2.5	32	1.3	46.6	7.4	20.4
16	14.2	56.7	1.38	6.32	1.82	4.07	252	1	4.4	4.9	38.1
16	15.3	83.3	1.96	5.78	1.01	3.81	227	2.8	12.1	15	1.9
25	13.6	60.6	0.83	5.68	1.97	3	605	1.8	10.3	9.2	5.3
32	18.1	59.4	1.45	3.97	1.09	2.19	166	1.3	4.8	6.9	7.8
26	10.1	84.2	1.43	6.12	1.48	4.37	121	0.4	5.7	4.3	2.5
12	9.4	62.6	1.05	4.47	1.37	2.36	74	1.5	22.2	10.6	1

15	10.7	68	3.03	7.85	1.45	4.14	1193	3.7	6.8	24.3	2.7
11	12.8	89.7	1.34	4.66	0.96	3.05	209	2.55	6.8	14.8	21.85
17	16.3	62	0.83	5.16	1.33	3.41	135	1.4	6.8	5.3	41
32	19	69.8	0.91	5.95	2.25	3.47	38	2.4	14.9	22.8	4.8
16	11.4	69.6	1.76	7.81	1.15	2.885	107.5	1.8	13.8	9.9	1
8	8.6	53.8	0.68	4.69	1.92	2.3	177	1.1	14.1	12.6	9.9
22	21.5	83.7	0.67	3.63	1.27	1.96	13	3	12.5	8.9	5.8
9	10	69.2	1.08	6.99	1.39	4.58	594	3.5	10.9	5.5	1.7
19	7.9	57.2	1.18	4.24	1.13	2.26	120	1.2	9.5	6.2	0.9
68	23.6	83.1	0.87	5.12	0.99	3.36	79	4.8	14.8	10.8	0.8
14	14.9	64.9	1.76	6.6	1.81	4.07	196	3.2	14.5	0	2.76
17.5	15.8	67.8	1.705	6.3	1.565	3.65	129	3.5	14.3	6.55	4.7
21	16.7	70.7	1.65	6	1.32	3.23	62	3.8	14.1	13.1	6.7
19	9.7	67.1	1.03	4.92	1.66	2.66	509	2	22.75	10.9	4.1
8	4.8	66.4	1.04	4.85	1.39	2.79	35	1.4	31.4	10.2	1.5
13	13.2	80.6	1.11	6.47	2.21	3.38	120	1.9	7	6.6	1.8
13	29.5	63.5	3.46	3.89	0.93	1.51	55	2.1	9.8	16.3	0.9
11	12.6	68.8	1.36	5.11	1.4	2.88	227	1.6	9.3	12.45	1.95
9	8.6	76.8	0.68	4.63	1.49	2.58	246	1.1	8.8	8.6	3
9	7.9	64.8	0.64	3.64	1.42	2.03	36	2	10	12.4	1.5
30	16.8	87.9	1.68	6.26	1.31	4.31	103	4.8	6	26.6	1.3
23	14.7	58.9	1.07	4.77	1.4	2.99	421	1.5	7.9	5.8	1.9
19	13.4	60.2	0.67	3.79	1.44	1.93	46	2.7	9.2	14.1	1.5
10	14.6	82.7	0.82	5.92	1.84	3.09	170	4	10.5	13.4	1.1
11	9.1	74.2	0.71	5.46	1.21	3.53	91	1.3	5	7.5	1.9
14	10.6	77.9	1.08	5.43	1.74	3.39	163	2.7	6.5	11.1	1.3
11	13.4	57.3	0.93	2.46	0.98	1.12	33	0.7	8.3	7.1	0.8
25	11.4	56.1	0.72	5.15	1.39	3.14	58	1.6	9.8	17.2	28.5
10	10.4	60.6	0.72	4.39	1.27	2.74	441	1.2	17.9	10.7	2.5
13	14.6	78.7	1.03	6.6	2.1	3.77	190	2.8	8.1	9	2.2
24	19.4	63.6	2.13	6.46	1.46	4.18	59	1.3	7.8	11.6	1
47	15	67	2.2	5.67	1.07	3.58	177	0.9	5.7	7.7	1.6
11	17.1	77.1	0.97	4.48	1.13	2.66	46	3	8.1	5.3	1.2
17	10.9	62.5	0.7	3.8	1.49	1.85	79	3.1	8.9	15.3	1.6
11	8.3	63.3	0.81	3.99	1.64	1.82	85	3.2	23.2	7.1	2.3
18	9.6	70.9	2.31	5.15	0.91	2.93	123	0.6	12.7	6.1	0.9
14	10.1	94.8	1.22	5.07	0.94	3.29	595	1	12.8	5.4	2.5
17	6.1	73.4	2.16	4.93	0.87	2.55	653	1.5	22.3	0	1.6
24	21.4	71	0.87	5.13	2.01	2.31	182	1.5	5.6	17.6	1
11	11.5	63.8	2.72	6.07	1.41	3.06	240	1.5	6.6	0	1.4
42	12.9	61.1	3.16	6.11	1.1	3.76	88	1.2	7.2	6.4	1.3
10	10.4	73.7	1.18	3.75	1.12	2.39	35	5.5	7.8	27.5	1.2
35	18.9	77.6	2.06	3.92	1	1.93	16	1.3	5.9	7.2	2.5
10	8.5	84.8	1.46	5.74	1.65	3.09	32	0.3	27.4	5.7	3.5
10	26	79.2	1.18	4.15	1.1	2.24	51	0.95	19.75	11.2	2.25
15	9.1	59.8	1.11	4.32	1.1	2.81	83	1.6	12.1	16.7	1
44	19.8	98.4	2.56	5.78	0.98	3.77	337	1.2	4.8	6	1.1
15	15.5	69.3	1.23	4.65	1.13	2.45	179	2.8	10	13.9	0.8
20	12.9	84.5	3.87	4.9	0.95	1.91	233	1.75	11.2	10.25	0.8
8	11.1	76.2	0.81	3.98	1.49	1.9	185	0.7	12.4	6.6	0.8
10.5	13.9	67	0.89	3.58	1.39	1.745	153.5	1.05	8.75	7.6	0.95
13	16.7	57.8	0.97	3.18	1.29	1.59	122	1.4	5.1	8.6	1.1
16	16.3	79.6	1.05	5.49	1.47	3.48	40	3.3	12.2	11.1	1.2
11	23.1	71.9	1.06	5.7	1.42	4.02	1062	1	5.2	9.8	18.5

18	14.8	78.2	2.07	3.72	1.16	1.67	214	1.4	19.9	6.7	1.9
16	12.4	59.5	3.26	6.64	1.13	3.73	153	2.3	11.4	10.2	1.3
13	17.4	64.4	0.99	5.37	1.33	3.48	93	0.9	10.4	8.5	1.9
16	18.1	85.8	3.09	4.41	0.9	2.24	105	1.3	6.2	3.3	1
13	6.5	67.7	0.57	3.81	1.28	2.21	24	0.9	5.8	4.7	14.4
12	12.1	71.4	0.61	4.29	1.46	2.41	36	1.2	13.2	16.4	3
18	7.2	65.4	0.85	3.98	1.3	2.33	58	1.1	13.4	8.6	1.1
10	14.8	62	1.58	4.72	1.12	2.71	103	0.6	17.5	13.8	1.5
8	13.8	68.3	0.88	4.61	1.61	2.38	66	1.3	11.2	9.8	0.5
15	7.4	62.2	1.15	5.17	1.67	2.92	410	1.1	11.2	3.5	1
13	10.1	67.9	0.7	6.38	1.63	3.89	460	0.5	5.6	18.3	0.9
19	12.5	73.8	0.8	2.98	0.96	1.7	40	1.9	4.7	8	1.4
17	14.5	78.8	0.54	5.19	1.9	3.13	71	2.4	4.4	0	2.1
16	10	89.6	2.35	4.54	0.66	2.71	54	2.7	10.6	18.1	1.1
13	12.4	51.6	0.84	4.95	2.48	1.61	74	2.9	23.3	5.4	1.5
19	11.4	77.6	1.42	4.295	1.69	1.685	82	1.8	17.3	6.65	3.6
25	10.4	103.6	2	3.64	0.9	1.76	90	0.7	11.3	7.9	5.7
14	6.4	51.4	0.93	4.09	1.44	2.2	83	1.4	14.9	9.1	0.7
9	14.3	59.8	0.9	5.07	1.7	2.81	1419	1.3	24.6	11.6	1.45
12	11.9	47.7	0.86	4.82	1.41	3.07	437	0.6	34.3	13.3	2.2
10	5.2	57.3	1.37	5.47	1.31	3.2	92	1	18.5	15.8	1
16	11.6	72	0.95	4.4	1.4	2.56	51	2.3	10.4	6.9	1.4
16	12.9	82.4	0.51	3.17	1.18	1.66	26	0.3	5.8	5.8	1.6
13	9.3	77.1	1.4	5.77	1.29	3.4	189	1.4	9.3	5.6	0.8
16	12	64.6	2.18	6.63	1.56	3.97	325	1.4	9.4	14	3.6
9	15.7	41.9	0.79	4.72	1.48	2.52	79	1.8	12.4	9.2	2.1
10	5.6	64.8	1.25	4.02	1.32	2.18	122	0.4	12.4	11	34.4
8	7.9	81.2	0.97	3.27	1.04	1.86	19	0.7	16.2	6.3	0.8
9	11.4	50.1	1.3	3.84	0.99	2.19	36	0.5	6.6	12.3	0.8
40	7.1	66.4	1.82	5.93	1	3.99	202	0.8	21.6	9.3	1.2
14	17	87	0.94	3.82	1.07	2.11	21	1.5	12.3	4.4	0.8
17	15.6	62.5	1.22	5.23	1.05	3.29	204	1.1	11.2	0	11
21	11.2	60.5	1.24	5.15	1.58	2.83	1588	1.1	11.1	14.3	1.3
17	9.4	61	1.5	4.535	1.295	2.54	841	1.1	11.25	10.3	2.1
13	7.6	61.5	1.76	3.92	1.01	2.25	94	1.1	11.4	6.3	2.9
15	18	77.7	1.44	4.18	0.93	2.46	60	2.1	10.2	17.9	1
19	25.3	76.3	0.85	5.08	1.51	3.02	457	2.9	5.6	13.8	1.2
9	13	58	0.82	5.37	1.53	3.25	46	2.1	12.1	27	1.6
28	17.4	93.6	1.49	4.31	1.36	2.95	60.5	1.05	15.65	18.7	1.55
9	10.8	59.4	1.42	5.59	1.41	3.27	75	0	19.2	10.4	1.5
22	13.5	62.9	1.72	3.66	1.07	1.83	174	0.5	13.5	8.15	1.2
23	10.5	69	1.16	4.47	1.61	2.26	96	1	7.8	5.9	0.9
13	6.1	65.1	1.1	2.35	0.6	1.44	263	1.8	5.3	0.9	0.8
16	7.3	130.4	1.15	2.7	0.54	1.94	266	1.2	10.65	2	1
11	7.8	83.3	1	4.18	1.11	2.62	92	4.8	16	21.8	1.2
14	6.7	93.3	1.65	6.94	1.11	4.62	647	2.3	436.4	128.4	7.1
10	14.3	61.9	0.58	4.45	1.5	2.6	720	5.3	9.1	0.7	3.7
13	8.4	91.9	1.43	4.67	1.08	2.74	388	2.4	5.8	6.5	1.6
12	7.7	108.9	1.04	4.53	0.99	3.05	135	113.6	10.1	132.3	3.3
44	7.8	78.5	0.71	3.55	0.85	2.31	431	1.5	18.4	45.6	4
14	7.2	56.8	1.33	4.88	1.12	3	245	18.3	59.5	718.1	7.8
8	9.7	61	0.54	5.32	1.8	2.94	327	0.8	15.9	5.2	3.8
27	12.9	96.8	1.14	3.81	1.22	2.03	149	2	12.2	109.3	2.15
15	9.3	57.8	0.93	3.5	1.28	1.72	37	2.7	8.5	17.8	0.5

38	9.1	70.8	4.26	4.89	0.89	2.58	101	2	19	12.3	4.5
20	26.8	105.6	0.62	4.59	1.42	2.8	334	3.4	7	1	11.2
11	10.7	81.4	1.63	4.37	1.15	2.48	244	48.8	14.4	75.5	16.8
22	15.2	71	1.54	5.25	1.03	3.17	291	0.7	11.6	9.1	5.7
6	17.3	94.9	0.84	4.65	1.26	2.79	618	8.4	10	31.6	1.1
23	10.5	68.8	0.87	3.63	0.99	1.95	258	3.6	10.4	12.9	1.1
9	21.2	80.1	0.94	3.54	1.22	1.78	128	2.1	12.5	13.8	0.8
13	8.7	81.5	1.22	5.01	1.01	3.22	188	0.7	10.5	6.7	4.2
11	7.9	61.3	1.43	4.63	0.78	2.93	114	1.8	13.5	1.9	0.8
6	14	58.1	1.13	6.31	1.55	3.97	58	1.9	7.8	1.7	1.5
15	14	107.6	0.97	4.01	1.12	2.36	94	2	21.7	6.6	19
24	11.3	72	0.62	3.07	1.26	1.32	458	3.5	8.3	1.6	16.1
22	19.7	74.3	0.58	3.71	1.37	1.81	31	2.8	5.3	26	1.1
34	17.1	64.4	1.34	4.97	1.54	2.66	207	0.8	32.3	23.2	0.8
11	9.9	72.9	0.9	4.81	1.27	2.94	126	0.6	12.5	9.7	0.7
24	23.3	76.9	0.72	3.39	0.58	2.3	96	1.5	297.9	59.9	9.4
10	9.9	69	1.06	4.33	1.25	2.46	330	0.6	13.3	9.3	0.7
14	9.7	104.1	0.74	2.88	1.15	1.48	116	3.7	48.8	11.5	0.7
30	16.4	69.9	1.76	3.1	1.31	1.37	45	2.3	31.25	15.7	5.75
29	6.3	61.9	0.61	3.92	1.84	1.79	132	1.7	13.7	7.5	10.8
33	12.9	90	4.2	4.3	0.72	2.27	49	1.8	15.9	12.6	7.5
15	12.4	83.8	1.45	3.36	0.79	2.03	113	2.3	6.1	7.3	2.3
9	11.8	83.8	0.72	2.75	0.74	1.71	51	2.3	7.1	8.5	6
10	9.7	86.2	1.14	4.3	0.92	3.38	76	1.3	7.5	5.4	0.7
31	10	69.1	0.93	2.7	0.77	1.53	101	36.5	17	353.9	2.8
11	7.2	68.5	0.56	3.32	1.12	1.85	20	0.5	5.2	8.3	4.3
15	14.3	76	1.11	5.28	0.97	3.52	185	5.7	6.95	20.1	10.35
18	5.1	88.2	1.32	2.66	0.74	1.34	64	50.3	8.7	28.3	16.4
10	16.5	82.8	1.14	3.09	0.93	1.6	274	1.2	45.4	5.3	0.9
14	15.8	81.3	1.68	3.82	1.08	2.24	198	1.4	4.8	8.4	7.9
22	10.3	90.7	1.18	3	1.02	1.54	113	5.2	19.6	22.2	1.5
19	9.5	92.8	1.42	3.71	0.79	2.39	129	2.5	8.4	1	9.2
20	15.2	67.7	1.12	5.13	1.51	2.81	51	0.9	37.25	16.9	5.85
9	7	52.6	1.66	4.73	1.4	2.71	1208	0.5	66.1	5.7	2.5
15	7	68.5	3.05	5.34	1.02	3.33	182	0.6	5.6	10	4.6
21	9.2	46	2.29	6.65	1.38	4.06	1108	0.5	15	5.7	2.3
24	15.9	97.4	1.49	4.36	0.94	2.71	572	171.6	9.9	21	7.5
13	9.1	65.6	1.21	3.35	0.97	1.85	253	7.8	19.5	143.5	127.6
10	13.7	69.8	1.47	4.18	1.29	2.17	23	1.3	19.3	10.8	2.6
11	8.1	73.1	2.57	4.28	1.15	2.02	141	1.5	93.9	11.5	3.6
11	11	62	1	5.45	1.27	3.6	41	1.8	28.1	1.2	49.8
13	8.7	71.3	1.24	3.22	0.75	2.03	110	5	139.3	2.4	1
23	15.4	78.5	2.18	3.94	0.59	2.35	37	4.1	30.6	62.5	3.1
10	7.2	66.4	1.01	4.31	0.72	2.95	71	2	21.15	16.9	2
97	23.6	89.2	0.84	4.14	1.26	2.4	160	2.7	11.7	1.3	0.9
11	10.4	71	1.49	6.35	1.41	3.89	367	1.2	16.3	59.5	4.8
20	9.2	61.1	1.34	4.62	1.21	2.66	457	2.9	14.4	16.8	1.6
9	8.2	65.2	0.6	3.66	1.6	1.44	633	2.4	8.6	12.55	2.2
8	11.7	58	1.2	4.76	1.28	2.72	328	1.9	2.8	8.3	2.8
17	22.1	87.8	0.53	2.13	0.79	1.04	136	2	25.7	11	0.8
17	6	115.3	1.17	4.23	0.78	2.69	1026	3.9	15.4	0.8	6.9
8	20.2	73.6	1.41	5.32	1.36	3.37	318	9.9	19.9	684.2	3.2
19	6.4	78.9	1.39	3.74	1.11	2.19	85	4.1	19.4	13.9	1.8
11	10.9	94.8	0.61	3.91	1.52	1.68	195	1.8	11.4	36.9	77.9

9	12.5	94.7	0.82	3.35	1.01	1.87	453	1.4	103.5	4.6	0
16	15.4	88	2.54	4.99	1.14	2.63	465	3.1	29	29.6	1.6
21	10.7	70.1	1.06	5.02	1.81	2.34	689	2.3	9.4	12.1	31.2
15	14	80.4	0.79	4.25	0.98	2.62	303	2.4	16.1	7.4	1.8
9	9	62	0.48	3.77	1.45	1.92	92	1.2	14.8	11.9	1
8	7.4	71.5	1.02	3.37	0.9	2.1	159	0.7	5.6	3.7	8.3
24	9.2	69.1	1.16	5.31	1.02	3.21	182	1	7.4	6.6	6.3
16	11.6	94.2	0.98	4.97	1.12	3.15	220	1.9	8.6	0.8	2.2
15	4.8	97	1.31	3.49	0.8	2.69	156.5	10.7	30.15	4.6	6.9
15	14.5	105.4	1.11	7.73	1.31	5.2	93	2.3	51.7	93.7	11.6
11	10.3	74.5	1.35	4.9	1.04	2.93	155	1	10.9	9	3.1
10	14.5	79.7	0.57	4.8	1.32	2.99	143	1.6	11.3	4.1	4.6
7	5.7	105.1	2.06	5.59	1.13	3.35	76	2.6	11.7	94.4	6.1
16	12.3	99	1.13	3.69	0.81	2.24	17	7.2	244.9	39.7	0.9
9	7.3	93	0.83	4.32	1.18	2.38	314	5	9.9	75	1.2
15	10.8	75.8	1.44	3.75	0.8	2.32	109	2.1	5.6	18.8	1
45	109.6	223.6	1.21	3.77	0.06	3.36	32	4.7	600.8	20.9	2.4
22	11.6	93.5	1.8	5.91	1.39	3.21	414	2.2	13.3	6.5	5.2
13	14.9	72.2	1.24	3.22	0.69	2.08	241	8	18.4	34	2.1
19	16.4	79.3	0.79	4.79	1.55	2.58	18	3.9	14.1	11.8	1.5
10	17.3	66.5	0.78	3.03	0.7	2.33	53.5	2.6	270.1	23.5	1
8	15.7	73.8	0.54	2.57	0.93	1.27	89	3.1	186.9	17.3	2.7667
16	12.5	106.9	0.95	4.74	1.28	2.92	48	6.3	103.7	1	4.5333
7	6.6	54.8	0.97	4.59	1.35	2.52	358	38	20.5	30.9	6.3
6	6.6	61.3	2	5.35	1.14	3.12	45	1.3	22.3	10.9	40.2
13	35.6	59.9	0.93	4.72	1.24	2.77	27	5.9	9.6	12.5	0.8
29	8.2	56.8	1.33	4.88	1.12	3	245	18.3	59.5	718.1	7.8
13	14.9	88.4	1	3.61	0.82	2.16	121	4.5	46.4	0.9	8.1
16	22	59.2	0.9	4.91	1.73	2.41	24	1.6	8.1	7.2	0.5
15	15.1	68.7	1.2	4.58	1.3	2.65	152	1	16.2	26.1	2.1
48	18.8	82.4	2.02	5.35	0.77	3.53	56	2.3	13.6	0.9	9.1
12	11.2	73.1	1.11	4.66	1.25	2.53	97	5.1	60.3	7.4	4.3
11	14.9	89.2	0.82	6.13	1.52	3.74	673	4	10.4	19.2	1.2
9	5.5	94.7	1.07	3.04	1.01	1.53	90	1.4	8.9	5.6	1.5
25	15.8	109.8	2.09	5.37	1.05	3.2	163	3.8	7.2	1.2	4.5
19	7.6	130.1	3.39	5.26	0.71	2.84	560	2.5	25.8	7.1	22.9
13	7.2	82.5	0.91	4.82	1.4	2.79	231	26.2	20.65	19.5	12.15
18	11.6	70.7	0.79	5.12	1.62	2.95	112	2.5	15.5	1.1	1.4
24	5.9	73.5	0.76	3.43	1.04	2.02	188	1.3	7.2	9.7	1
12	35.3	84.9	0.65	4.63	1.59	2.62	17	2.7	7.3	5.8	1.7
20	9.2	61.1	1.34	4.62	1.21	2.66	457	2.9	14.4	16.8	1.6
16	14.9	98.8	0.64	3.09	0.89	1.93	110	37.2	19.7	213.4	45.7
14	11.1	66	1.32	6.04	1.52	3.69	453	1.4	11.8	1.1	1
10	9.7	86.2	1.14	4.3	0.92	3.38	318	1.3	7.5	5.4	0.7
11	11.5	55.9	0.95	4.49	1.05	2.86	183	1.4	7.4	11.2	1.7
16	20.6	108.4	1.94	5.34	1.15	3.12	251	2.1	12.2	23.2	4.2
6	14.5	87.2	0.83	5.84	1.49	3.45	689	2.4	6.4	11.2	3.8
14	18.7	71.2	1.42	3.38	0.97	1.61	536	3.6	7.3	20.1	0.9
13	8.4	91.9	1.43	4.67	1.08	2.74	388	2.4	5.8	6.5	1.6
22	15.2	71	1.54	5.25	1.03	3.17	291	0.7	11.6	9.1	5.7
30	11.2	70.6	1.65	5.5	1.94	2.88	73	1	7.1	5.9	1
9	10.9	80.2	1.66	3.15	1.03	2.12	87	0.8	11.3	7.1	1.7
38	9.1	70.8	4.26	4.89	0.89	2.58	101	2	19	12.3	4.5
8	8.7	113.2	2.13	4.37	0.87	2.66	341	146.9	133.4	7108	2.7

6	19	61.9	0.48	1.85	0.41	1.21	99	13.8	247.8	26.3	0.9
14	21	93.6	0.92	4.25	1.12	2.51	76	1.8	9.7	9.1	1.3
22	9.1	103.9	1.88	5.52	1.11	3.45	971	2.4	14.45	7.4	3.4
24	11.6	67.5	2.41	7.11	1.27	4.8	15	1.9	19.2	9.2	5.5
31	11	63.8	1.24	3.38	0.87	1.98	156	0.8	32.3	23.2	0.8
8	5.5	77.6	1.13	2.93	0.71	1.64	284	4.3	15.9	670.4	8.6
8	26.8	65.3	0.62	3	0.92	1.62	13	1.2	6.2	10.1	0.6
17	16.2	80.9	1.97	4.51	0.84	2.73	22	1.2	16.1	14.4	3.9
12	13.6	84.7	1.65	6.6	1.41	4.14	693	1.2	8.1	7.6	13.1
22	12.9	65	0.92	4.77	1.51	2.52	189	2.5	12.6	1.9	0.9
15	17.5	64.6	3.9	5.18	0.99	2.15	113	2.3	80.7	14.3	3
15	14.9	107.6	0.97	4.01	1.12	2.36	94	2	21.7	6.6	19
17	10.6	83.9	0.92	2.88	0.94	1.41	84	1.7	10.9	9.1	9.3
11	11.8	91.3	1.71	3.51	1.21	1.34	131	2.7	11.15	195	12.95
21	14.6	57.2	1.39	5.93	2.19	2.53	165	4.3	11.4	23	16.6
8	15.5	78.7	0.68	3.84	0.93	2.11	112	3.5	9.4	32.8	1.2
12	17.2	82.8	1.69	3.38	0.86	1.66	25	1.1	25.7	6.1	1.3
27	6.2	61.8	1.58	4.18	0.97	2.39	170.3	2	6.2	4.5	1
19	24.2	82.7	1.47	4.98	1	3.13	315.6	2.15	9.55	6.7	3.3
11	42.2	103.6	1.36	5.79	1.2	3.87	461	2.3	12.9	8.9	5.6
60	18.9	82.8	1.2	4.73	0.81	3.18	30	3.7	10.4	6	1.4
11	11	62	1	5.45	1.27	3.6	41	1.8	28.1	1.2	49.8
20	19.3	55.4	1.68	6.22	1.37	3.91	53	0.5	13.4	20.1	1.1
11	8.1	73.1	2.57	4.28	1.15	2.02	141	3.8	12.1	19.1	2.7
25	10	80.3	0.86	6.17	1.02	4.14	322	2.8	6.6	10	7.2
11	8.4	86.8	0.69	4.81	0.86	3.08	152	4.2	10	1.3	4.35
12	5.5	63.4	1.55	2.57	0.68	1.25	169	18.4	13.4	100.4	1.5
19	14.5	77	1.38	5.98	1.4	3.39	354	1.7	16.9	11.8	2
13	16.4	58.2	0.93	5.29	1.55	2.86	28	2	8.7	16.6	1
7	17.2	68.4	0.99	4.98	1.25	3.17	27	171.5	1083	8.1	54.7
24	12.8	65.4	1.39	5.53	1.58	2.81	262	1.7	725.5	6	36.8
69	25.3	73	0.8	4.59	2.18	1.88	231	3	368	7.6	18.9
11	6.2	49.6	0.92	4.2	0.97	3.23	133	2.5	10.6	17.9	1.1
11	15.5	84.4	1.02	4.96	1.16	2.98	35	1.5	7.6	8.1	1.7
8	13.9	94	1.35	3.55	1	1.93	926	0.3	6.9	39.2	3.7
36	23.4	72.6	0.55	4.48	0.99	3.06	144	116.6	181.1	0	15.5
25	15.7	70.8	0.99	3.9	1.19	2.2	46	2.2	14.3	6.3	1.2
15	8.9	86.4	1.62	4.07	0.79	2.48	332	24.9	12	243.5	271.9
12	1.4	73.7	0.98	3.51	1.04	1.76	108	22.4	18.4	114.9	3.6
38	15.2	112.9	1.05	3.12	0.68	1.86	259	2.7	5.4	19.9	0.8
10	20.1	86	1.84	4.31	0.9	2.58	155	2.8	16.9	8.9	1.5
5	9.9	63.9	1.24	2.55	0.71	1.59	333	154.6	82.1	180.4	52.1
8	4.9	76.8	2.7	2.3	0.55	1.12	75	79.4	46.15	6.6	26.95
20	15.3	76.8	1.33	3.9	0.8	2.58	490	3.1	10.2	8.5	1.8
13	7.2	70.9	1.07	4.43	1.05	2.58	413	0.9	12.2	0	9.6
3	8.1	59.5	0.83	3.31	0.83	2.01	48	1.4	7.3	58.1	1.9
16	10.6	80.8	1.52	5.27	0.88	3.95	113	3.1	12.2	37.5	9.4
21	13.1	108.3	1.86	3.84	0.88	2.59	28	6.3	9.7	27.8	1.6
10	8.1	65.5	0.63	4.3	1.1	2.71	177	5.7	76.2	0	1.35
9	21.9	53.6	1.36	2.55	0.51	1.48	182	8.2	142.7	287.5	1.1
15	8.6	73	1.05	3.4	0.72	1.95	89	3.5	12	107.8	21.6
41	8.2	62	0.97	5.32	1.71	2.67	46	2.5	19.6	8.1	1.4
10	11.4	63.5	0.72	2.71	1.04	1.25	343	3.3	11.1	26.6	1.1
31	12	97.9	1.36	3.87	1.19	1.89	56	133.9	15.2	38.5	49.7

12	9.1	74.3	1.3	4.79	1.09	2.81	132	2.2	12.6	6.1	3
16	13.1	68.3	1.55	5.33	1.77	2.5	20	3	17.1	294.2	1.9
13	28.1	77.8	1.16	4.13	1.23	2.55	70	5.9	7.2	8.2	2.8
9	5.6	82.6	1.77	3.66	0.67	2.02	64	1.7	11.7	13.4	1
16	9.5	64.3	3.02	5.43	1.2	3.37	351	2.6	20.1	0	27.8
8	7	64.7	0.89	1.97	0.63	1.03	156	5.7	13.6	0	1.5
20	17.9	90.1	0.75	5.19	1.58	3.18	147	2.7	5.3	6.8	4.9
18	17.7	78.3	0.66	3.85	1.08	2.27	68	1.8	5.2	6.2	3.3
46	12.4	49.4	1.61	7.55	1.2	4.88	41	2.9	9.1	0	29.9
26	6.7	110.7	0.94	3.53	1.65	1.09	118	6.2	18.1	40.7	5.5
9	12.4	77.3	0.73	5.09	1.51	2.69	222	5.9	19.2	119.7	2.3
18	10.8	579.6	0.72	3.63	1.09	2.18	269	6.4	43.7	13.1	18.9
14	10.7	95.4	0.8	3.83	0.99	2.74	326	2.3	10.3	0	1.9
17	11.3	62.6	1.64	4.26	1.04	2.4	85	0.9	11.4	4.5	1.8
14	16	79.7	1.07	6	1.41	3.69	101	92.5	17.6	18.3	7.9
21	13.6	81.3	0.89	3.49	1.22	1.8	50	3.2	10.75	4.8	4.9
15	6.4	72.2	0.46	3.47	1.26	1.97	72	0.8	3.9	10.8	1.9
15	9.5	63	1.42	3.91	0.9	1.7	52	6.9	10.3	70.6	1.3
15	6.6	65.6	1.42	4.54	0.99	2.64	177	6.2	10.45	11.8	1.4
14	8	119.4	0.66	3.89	0.88	2.58	134	5.4	10.6	34.4	1.5
8	20.2	73.6	1.41	5.32	1.36	3.37	318	9.9	19.9	684.2	3.2
16	9.5	83.1	0.95	3.3	0.79	2.13	107	1129	28.6	42.3	5.1
56	13.4	64	1.41	4.64	1.24	2.78	78	3.5	7.3	11.9	1
11	5.7	76.3	0.82	3.58	0.64	2.29	505	1.7	82.1	0	0.8
6	9.7	70.2	0.93	4.6	0.99	1.91	376	5.9	94	3218	52.8
8	6.9	68.8	0.84	3.48	0.89	2.31	1074	1	30.3	5.2	1.4
8	8	80.9	1	3.18	1.01	1.62	86	1.2	37.9	8.8	1.7
17	15.1	80.7	0.86	4.63	1.5	2.58	170	3.9	76.9	16.5	0.8
17	14.7	79.2	0.97	3.93	0.74	2.56	127	1.9	34.4	21.4	2.9
17	10.4	85.5	0.99	3.87	0.98	2.43	735	1.5	3	8.3	1.1
11	7.5	81.5	0.96	6.73	1.38	4.97	419	2.4	10.6	6.9	6
11	11.8	131.9	1.24	3.58	0.88	2.35	51	1.6	7.3	0	1.3
31	9.7	50.3	0.85	4.46	0.9	2.96	94	1.3	55.3	24786	19.26
16	12.7	66.8	0.82	3.07	0.73	2.01	90	2	11.4	5.7	1.4
21	26.2	77.4	0.78	4.79	1.54	2.94	62	6.9	39.85	11.6	128.8
27	7.4	50.8	1.58	4.44	1.15	2.52	59	277.9	68.3	1346	256.2
10	7.2	71	1.25	5.12	0.99	3.62	54	1.9	9.4	6.5	23.2
12	13.7	59.2	0.49	4.32	2	1.9	964	1.8	9.9	0	13
15	14.6	57.8	2.07	6.15	1.25	4.08	101	1.5	10.4	0	2.8
16	10.9	71.7	0.96	5.18	1.18	3.55	146	2	8.25	18.8	2.5
23	7	74.7	2.14	4.75	0.92	2.86	41	3.7	6.1	2.6	2.2
18	16.7	82.6	0.88	3.87	1.51	1.78	47	3.7	5.2	11.2	3.5
12	10.4	79.5	0.69	4.91	1.43	2.94	358	1.5	10.8	4.7	0.7
26	19.3	90.2	0.82	2.87	1.07	1.36	54	2.8	19.7	464.2	1.6
8	11.5	76.9	0.77	3.91	1.12	2.29	125	2.3	8.3	25.7	1.1
155	15.1	77.3	0.42	2.48	1.07	1.34	203	2.5	9.5	4.9	28.1
21	15.8	55.7	1.15	4.8	1.14	3.09	241	0.7	8.5	8.1	10.9
15	9.2	77	1.66	4.37	1.04	2.8	124	2.8	16.1	7.4	2.2
10	15.6	89.2	0.74	4.14	1.05	2.47	97	2.2	5.2	15.6	1.9
16	18.6	95.8	2.4	3.91	0.87	2.08	52	2.1	8.6	6.9	1.5
12	9.7	68.8	0.78	6.66	2.1	3.98	71	0.5	10.4	9.9	0.7
6	17.3	94.9	0.84	4.65	1.26	2.79	618	8.4	10	31.6	1.1
19	10.1	58.1	0.7	2.44	0.69	1.26	106	5.8	50.8	21.3	37.9
18	7.8	68.2	0.78	4.1	0.65	2.98	91	1	10.6	19.6	0.7

13	11.3	62.4	0.78	6.21	1.77	4.27	41	1.5	13.2	8.3	10.1
8	10.6	68.1	0.96	2.96	0.59	1.76	189	2.9	33.4	197.7	5.3
9	17.5	86.8	7.25	4.24	0.81	2.48	169	7.4	5.9	21.3	2.8
24	4.6	94.8	0.97	4.37	1.19	2.7	1204	1.6	24.1	66.6	2.8
11	21.1	60	1.09	4.09	1.31	2.36	227	2.2	9.8	9.8	2.2
14	19.8	70.9	1.21	3.93	1.21	2.18	174	3.3	9.6	0	1.1
12	20.1	54	0.75	5	1.21	3.01	178	0.6	10.9	23.5	0.8
19	11.4	82.8	1.19	3.55	0.91	1.89	336	1.4	16.8	18.5	2.1
31	11.1	80.2	0.83	4.2	1.3	2.34	91	1.35	15.26	13.4	1.8
19	11.9	79.4	1.23	4.84	1.13	3.07	75	1.3	13.7	8.3	1.5
13	11.5	64.9	1.63	5.43	1.23	3.18	101	2.6	12.2	124.7	1.2
26	9.7	54.8	1.32	5.81	1.5	3.32	155	0.6	9.4	3.8	3.5
41	10.3	62.4	1.79	5.34	1.27	3.38	291	3.6	15.5	0	1.7
87	228	65.7	2.41	7.84	0.51	5.95	278	4.8	14.35	164.6	5.9
12	12.3	81.1	1.17	5.48	1.44	3.21	172	2.2	13.2	23.6	10.1
34	13.8	97.2	1.77	4.68	0.96	2.79	65	5.3	20.9	18.7	1.3
24	14.8	84	1.95	6.53	1.28	4.04	219	59.1	10.7	6.8	8.1
12	10.4	81.3	1.85	3.83	0.89	2.04	33	1.5	12.4	4.7	1.7
18	11.6	95.3	2.08	4.6	0.81	2.68	195	2.2	6.4	7	1.1
26	17.5	74.4	0.75	4.36	1.56	2.16	54	3	9.6	13.7	4.5
12	8.2	74.3	0.94	4.91	1.24	3.08	372	4.8	16	10.7	0.9
19	13.5	81.3	0.78	4.84	1.49	2.68	182	3.4	13.1	7.2	1.06
11	12.3	90.1	1.55	3.67	0.83	2.25	174	3	10.26	0	1.23
13	11.8	121.4	1.91	3.99	0.64	2.36	39	1.9	7.4	7.6	1.4
14	9.8	66.6	0.63	5.23	1.75	2.89	282	2.3	23.8	0	2
9	10.8	125.2	0.93	4.15	0.95	2.56	83	14.1	30.1	39.1	0.9
16	13.1	75.5	1.87	2.9	0.7	1.35	266	323	32.6	0	15.2
10	13.4	72.2	1.1	3.4	1.19	1.78	63	2.6	8.4	20.2	1.4
23	6.2	91.2	0.92	3.12	0.83	1.81	364	1.9	46.4	2.9	1.1
7	23.8	72.2	1.11	3.21	0.73	2.37	37	1.9	7.4	26.9	1.9
7	13.5	56.9	0.82	3.47	1.17	1.87	130	2.2	58.6	39.1	6.3
14	20.6	57.9	0.69	5.09	1.59	3.11	298	2.4	20.9	8.2	4.9
7	10.9	59.8	1.09	3.55	0.82	2.15	72	8.4	11.5	4.1	53.6
24	11.5	60.5	4.93	7.12	1.11	3.22	172	1	8.8	17.4	0.8
13	18.4	83.9	0.79	3.83	1.22	2.3	132	5.5	12.2	0	10.2
24	4.4	56	1.16	5.77	1.22	4.03	1795	1.5	41.4	2.4	12.4
15	9.2	113.5	0.4	2.28	0.67	1.38	170	4.5	20.7	5.9	4.4
6	4.8	80.3	0.92	4.49	0.9	2.82	521	20	11	543.8	5.8
9	12.5	65.7	1.03	6.93	1.19	4.9	376	1	469.4	9.1	1.4
8	6.6	80.7	1.29	4.97	1.11	3.19	141	1.2	7.2	27.7	1.2
9	16.7	90.5	1.3	6.41	1.29	4.17	135	1	10.9	7.6	1.2
14	8.1	135.5	2.51	4.65	0.76	2.66	317	0.6	210	6	129.8
24	6.9	62.1	1.7	6.24	1.31	3.64	73	1.2	3.8	11	11
18	19.4	68.6	1.06	2.23	0.82	0.79	67	1.6	4.8	10	6.5
16	9.7	86.9	3.35	3.24	0.75	1.73	92	2	5.8	9	2
9	10.6	64.7	0.55	3.63	1.05	2.37	67	4.2	12.8	12.5	6.3
14	15	59.1	1.22	6.35	1.24	4.01	349	5.3	13	14.1	2
23	10.6	47.8	1.95	5.51	1.03	3.76	95	12.8	451.7	178.6	600
11	14.3	69.5	1.57	3.11	1.04	1.09	40	0.7	8.1	3.8	2.1
6	9.2	60.8	0.42	3.17	1.37	1.5	186	8.3	13.6	0	0.9
8	12.2	62.1	0.71	3.96	0.96	2.44	259	9.7	110.4	13.2	2.3
15	14.2	95.6	1.66	4.35	0.97	2.82	60	2.9	7.4	15.1	1.3
37	18.7	96.9	0.67	4.93	1.51	2.54	739	1.1	6.6	1.7	7.6
162	77.6	82.5	1.24	5.92	1.67	3.31	58	36.2	8.2	0	89.2

25	16	88.1	0.5	3.93	1.55	1.91	662	2.9	15.9	9.1	11.1
19	7.5	73.5	1.83	5.37	0.96	3.18	63	9.4	35	32.8	2.7
14	30.5	82.4	0.64	4.98	1.24	3.63	183	1.8	33.1	53	2.3
16	11.1	98.7	1.01	4.49	1.19	2.64	407	100	306.9	348.7	3.8
8	13.8	76.8	0.74	5.63	1.33	3.27	1010	1	5.7	8.4	1.1
10	14.1	77.9	0.55	3.71	1.45	1.85	128	1.4	8.7	13.8	1
28	15	81.9	0.81	4.52	1.11	2.84	103	3.1	7.6	0	1.5
22	16.2	80.2	1.08	4.79	1.45	2.72	211	1.3	4.6	8.8	7.1
14	36.5	120	0.59	2.86	1.02	1.62	231	2.2	16.3	8.7	35.4
8	6.9	71.2	1.14	4.88	1.2	3.25	1174	4.2	79.4	15.6	1
14	38.5	105.2	1.35	2.51	0.8	1.3	175	4.1	53.95	7.7	1
12	8.9	73.4	1.01	3.48	0.73	2.25	110	3	28.5	3.2	1
13	9	97	1.03	5.22	1.21	3.42	106	2.7	9.8	6	0.8
20	9.4	78.1	1.02	3.86	0.8	2.48	276	2.9	19.9	745.9	3.9
11	18.7	71.7	0.95	4.33	1.75	2.04	165	1.2	10.7	10.6	2.3
177	230.6	87.3	2.89	7.54	0.43	6.07	27	3.3	46.1	98.5	20
14	20.2	89.2	1.7	3.77	1	2.03	26	9.7	8.2	29.9	5.3
10	5.4	55.1	2.59	3.87	1.23	1.98	123	2.5	17.8	25.3	6.7
18	11	58.6	0.71	3.12	1.14	1.61	519	4	19	39.1	3
13	16.3	89	1.71	6.53	0.79	4.66	130	20.6	8.3	16.6	39.5
15	10.4	71	1.01	5.2	1.55	3.14	275	1.7	10.9	6.9	1.1
7	8.1	65.1	1.44	4.55	1.15	2.64	100	2.5	70.7	14.2	5.9
11	9.1	65.2	1.41	6.08	1.28	4.15	155	2.8	8.7	26.9	2.5
20	12.5	77.9	1.16	5.62	1.75	3.04	133	1.1	6.8	9.2	1.9
11	12.4	99.6	1.18	3.63	0.93	2.16	151	4.1	4.1	11.9	0.9
7	7	81.1	1.02	2.8	0.58	1.86	488	1.2	13.3	113	11.6
15	12.1	89.9	1.09	4.64	1.23	2.79	129	3.4	5.8	18.8	0.9
7	9.2	73.9	1.33	2.76	0.68	1.5	264	1.5	14.1	5	9.8
11	28.9	73.9	1.2	5.2	1.25	3.17	283	2.1	5.6	4.8	1
15	9	58.3	0.79	3.35	0.79	1.96	435	1.5	544.1	855.8	55.2
16	11.4	71.3	0.98	3.54	1.14	1.81	216	5.4	21.3	21.3	1.3
13	7.8	103.2	0.43	3.74	1.34	1.99	87	1.9	8.5	7.3	1
25	9.3	101.1	1.16	4.02	1.28	2	261	1.6	227.25	14.4	2.75
65	38	90.1	2.12	7.07	0.41	5.86	326	1184	446	35.2	4.5
9	16.4	72.7	1.51	5.18	1.26	3.02	282	3.6	3.7	4.9	1.1
8	15	61.8	1.21	3.97	0.9	2.34	315	6.2	39.1	102.5	36
15	12.6	90.7	1.64	4.01	1.11	1.99	173	3.4	5.6	169.7	2.3
13	7.1	211	1.41	3.7	0.91	2.09	357	4.9	17.1	0	0.9
17	12.4	92.4	1.05	2.77	0.79	1.6	132	5.15	14.3	5.8	0.95
15	15.5	87.6	0.66	3.97	1.06	2.5	125	5.4	11.5	11.6	1
16	7.3	83.3	1.29	4.06	0.92	2.6	282	2.5	16.1	6	1.2
10	8.2	88	0.88	3.4	0.64	2.28	168	1.2	19.8	20.1	2.2
17	10.8	65.3	2.16	6.81	1.37	4.04	756	4.5	17.7	43.6	2.3
9	13.2	76.2	1.44	3.28	0.97	1.8	478	3.1	12.85	30.8	4.05
29	8.9	86.8	0.56	3.85	1.2	2.34	84	1.7	8	18	5.8
14	5.7	87.3	0.54	3.97	0.89	2.87	113	2.8	33.4	35	3.75
12	10.2	75	0.89	3.93	1.01	2.43	298	1.3	58.8	4.5	1.7
7	19.8	80	1.3	7.05	1.1	5.36	82	1.4	9.6	17.7	1
15	16.1	89.2	0.89	4.54	1.18	2.86	315	4.9	10.7	11.1	13.8
14	14.3	72.5	0.82	3.74	1.3	2.02	242	2.4	7.2	17.1	6
21	9.7	80.3	1.89	3.58	0.71	2.24	40	1.7	8.55	10.95	3.4
13	12.9	69.6	2.8	5.59	1.34	2.74	77	1	9.9	4.8	0.8
14	14.1	65.9	1.14	3.35	1.2	1.43	98	1.8	11	8.6	5.5
12	8.1	69.7	1.04	3.82	1.08	2.05	77	1.4	5.9	3.8	0.9

9	7.7	83.2	1.01	5.22	1.13	3.09	179	2.1	8.5	6.4	1.4
12	13.5	84.9	0.91	3.48	0.94	2.22	200	2.3	6	5.6	1.1
14	16.3	60.2	1.51	4.84	1.1	2.67	127	3	12.1	0	0.9
40	15	73.9	3.4	4.61	0.7	2.23	44	5.5	14.1	21.7	1
15	12.9	64.8	1.85	5.26	1.29	2.98	237	1.5	10.1	9.7	1.3
8	17	75.9	1.78	6.51	1.22	3.92	387	0.8	11.6	0	13.3
16	13.1	69.7	1.06	5.73	1.74	3.54	328	7.3	7.6	20.7	5
10	6.6	83.8	2.1	3.31	0.67	1.66	269	0.8	883.8	22.9	19.3
8	10.2	80.5	1.71	5.39	1.15	3.64	434	4.7	16.4	31.7	2.2
11	17.1	73.6	2.1	3.97	0.79	2.25	287	9.3	13	291.3	100.7
24	19.2	96.4	1.39	3.8	0.77	2.23	146	3.4	48.6	1589	11.7
9	10.6	102.7	1.4	6.78	1	4.28	408	7.8	7.3	10.6	1.8
18	11.9	61.4	1.7	4.26	1.3	1.83	102	3.2	18.9	0	15.4
14	19.6	90.6	1.81	5.05	0.88	3.07	60	2	6.7	17.4	1.1
12	7.8	84.1	0.93	2.89	0.79	1.69	23	12.9	13.3	25.3	10.1
19	11.4	91.7	1.74	5.18	1.36	3.04	44	8.9	10	38.8	1.7
12	10.8	57.2	1.24	5.02	1.5	2.92	90	3	8.7	13.9	1.2
17	15.4	94.9	1.35	4.87	1.21	2.79	135	0.8	5.7	4.2	1.8
8	13.6	97	1.44	3.22	0.49	2.15	403	1.6	28.3	9.2	1.4
9	7.7	94.9	1.08	5.28	1.27	3.17	304	3.5	74.8	4574	23.7
12	7.6	81.8	1.08	4.46	1.21	2.71	30	2.9	12.1	30.4	3.3
29	10	70.2	3.06	5.06	1.02	2.82	39	2.1	12.9	7.8	2
13	15.7	69.6	1.2	4.65	1.41	2.65	554	40.9	16.6	544.2	3.7
7	10.4	57.5	0.68	3.24	1.08	1.76	432	5	105.9	6.9	10.1
26	8.8	64.4	2.43	7.16	1.02	4.37	78	32	71.65	31.8	5.5
10	19.2	77.6	0.94	3.7	0.56	2.61	511	1.4	37.4	4.2	0.9
120	8.7	51.6	1.23	3.01	0.66	1.73	214	33.4	22.9	10.5	600
8	9.7	92.5	0.73	3.79	1.21	2.27	364	2	5.3	6.1	1.6
12	13.1	64.5	1.17	4.21	1.32	2.43	209	12.9	26	111.3	1.2
8	9.4	102.5	2.13	6.19	0.93	4.25	686	79	33.6	753.2	153
12	8.1	66.1	1.1	5	1.53	3.01	1426	2	23.35	63.5	283.5
14	8.2	72	1.26	3.64	1.03	1.84	99	4	13.1	75.9	414
17	17.5	113.4	1.49	5.52	0.9	3.81	307	1	17.2	25.1	1
7	17.2	83.1	1.15	3.94	0.98	2.36	135	1.2	14.6	3.8	1
27	8.1	76.9	0.77	4.36	1.04	2.68	103	3.2	10.2	26.6	70.9
60	10	70.1	0.81	3.32	1.2	1.6	46	19193	1906	148.5	33.4
122	16.6	94.8	1.55	2.83	0.7	1.59	41	1.4	10.9	9.5	0.7
28	10.5	99	1.79	5.71	1.36	3.23	59	2.5	4.3	8.1	1.3
13	8.4	79.8	1	3.98	0.64	3.03	357	3.3	16.6	5.4	1.7
24	14.6	74.7	1.48	5.64	0.87	4.05	29	2.1	7.6	14.4	1
14	9.5	85.3	1.73	4.05	0.87	2.4	303	1.3	9.8	66.5	5.2
12	16.3	86.9	1.85	5.3	1.33	3.19	193	2	12	19.2	9.4
12	8.2	46	1.68	4.73	0.96	2.83	36	1.9	11.1	7.4	1
12	4.3	51.9	0.99	3.6	1.04	1.99	35	16.9	230.3	290.3	2.6
15	15.4	95.1	0.92	4.33	1.16	2.49	315	1.4	7.3	6.6	1.3
35	14.3	79.3	1.94	5.39	1.1	4.29	239.5	2.1	8.6	1.4	14.8
37	20	71.5	1.42	7.09	1.42	4.64	164	1	7.7	7.7	8.7
11	14.3	51.5	1	3.36	1.16	1.73	189	1.4	6.8	8.5	2.6
10	14.8	71.3	2.24	4.78	0.95	2.83	80	1.7	6	12.4	1.3
12	11.8	67.6	1.77	4.17	1.04	2.21	146	1.4	11.5	13.7	1.9
8	16.1	101.8	1.31	5.13	1.03	3.21	214	0.8	20	6.2	0.8
24	10.1	82	1.9	2.93	1.02	1.15	30	3.2	8.4	15.5	1.1
6	14	58.1	1.13	6.31	1.55	3.97	58	1.9	7.8	1.7	1.5
12	34.5	93	1.34	6.67	1.38	4.46	28	1.6	10.8	103.1	6.4

17	17.9	73.7	1.31	5.76	0.9	3.75	249	4.4	22.4	0	83.5
14	17.9	66.5	1.72	5.36	1.46	2.87	961	2	10.2	11.8	1.3
9	11.3	77.1	1.16	5.79	1.6	3.7	439	3.8	16	119.4	1.3
22	24.9	66.2	1.44	4.95	1.06	3.17	175	476	6.2	632.8	10.9
16	9.4	56.9	0.82	7.24	1.73	4.47	359	0.8	30.4	5.2	7.8
24	24.6	83	1.75	3.3	0.97	1.67	42	0.6	9.6	0	1.5
26	7	69.8	0.74	2.11	0.91	0.78	89	26.3	5.9	8.6	7.9
14	9.3	90.1	0.87	4.75	1.2	2.97	191	1.8	107.5	4.5	2.4
